# The C4 protein encoded by tomato leaf curl Yunnan virus reverses transcriptional gene silencing by interacting with NbDRM2 and impairing its DNA-binding ability

**DOI:** 10.1371/journal.ppat.1008829

**Published:** 2020-10-01

**Authors:** Yuzhen Mei, Yaqin Wang, Fangfang Li, Xueping Zhou

**Affiliations:** 1 State Key Laboratory of Rice Biology, Institute of Biotechnology, Zhejiang University, Hangzhou, Zhejiang, China; 2 State Key Laboratory for Biology of Plant Diseases and Insect Pests, Institute of Plant Protection, Chinese Academy of Agricultural Sciences, Beijing, China; University of California, Davis Genome Center, UNITED STATES

## Abstract

In plants, cytosine DNA methylation is an efficient defense mechanism against geminiviruses, since methylation of the viral genome results in transcriptional gene silencing (TGS). As a counter-defense mechanism, geminiviruses encode viral proteins to suppress viral DNA methylation and TGS. However, the molecular mechanisms by which viral proteins contribute to TGS suppression remain incompletely understood. In this study, we found that the C4 protein encoded by tomato leaf curl Yunnan virus (TLCYnV) suppresses methylation of the viral genome through interacting with and impairing the DNA-binding ability of NbDRM2, a pivotal DNA methyltransferase in the methyl cycle. We show that NbDRM2 catalyzes the addition of methyl groups on specific cytosine sites of the viral genome, hence playing an important role in anti-viral defense. Underscoring the relevance of the C4-mediated suppression of NbDRM2 activity, plants infected by TLCYnV producing C4(S43A), a point mutant version of C4 unable to interact with NbDRM2, display milder symptoms and lower virus accumulation, concomitant with enhanced viral DNA methylation, than plants infected by wild-type TLCYnV. Expression of TLCYnV C4, but not of the NbDRM2-interaction compromised C4(S43A) mutant, in 16c-TGS *Nicotiana benthamiana* plants results in the recovery of GFP, a proxy for suppression of TGS. This study provides new insights into the molecular mechanisms by which geminiviruses suppress TGS, and uncovers a new viral strategy based on the inactivation of the methyltransferase NbDRM2.

## Author summary

Interfering with transcriptional gene silencing (TGS) is essential for DNA viruses to infect and replicate in plant cells. Some members of the *Geminiviridae* family encode a C4 protein capable of inhibiting TGS, hence promoting virulence. However, the molecular mechanism underlying the C4-mediated TGS suppression is still incompletely understood. This study demonstrates that the C4 protein encoded by tomato leaf curl Yunnan virus interacts with NbDRM2, a pivotal DNA methyltransferase in the methyl cycle, and interferes with its ability to bind the viral DNA. As a result, methylation of the viral genome is impaired, which in turn favours viral replication and spread. Our results uncover a novel strategy deployed by geminiviruses to counter anti-viral DNA methylation, hence shedding new light onto the arms race between viruses and hosts.

## Introduction

Cytosine DNA methylation is a conserved epigenetic mark in plants, animals, and fungi that modulates a number of biological activities including genome imprinting, transposon control, and transcriptional gene silencing (TGS) [1–4]. Importantly, DNA methylation also functions as a defense against invading DNA, such as geminiviruses [5–7].

Geminiviruses are a family of single-stranded DNA (ssDNA) viruses that cause devastating diseases in economically important crops worldwide [6, 8–13]. Geminiviruses are divided into 9 genera (*Becurtovirus*, *Begomovirus*, *Capulavirus*, *Curtovirus*, *Eragrovirus*, *Grablovirus*, *Mastrevirus*, *Topocuvirus*, and *Turncurtovirus*) based on genome structure, host range, and insect vector [14]. *Begomovirus* is the largest genus in the *Geminiviridae* family; viruses belonging to this genus are classified into two groups: monopartite begomoviruses, with genomes comprising one molecule only, and bipartite begomoviruses, with genomes comprising two molecules. Some begomoviruses in the New World, such as tomato golden mosaic virus (TGMV), are bipartite, having two separately encapsidated genome components, DNA A and DNA B. However, most begomoviruses from the Old World, such as tomato yellow leaf curl China virus (TYLCCNV), have a unique genome component, similar to the DNA A of the bipartite begomoviruses [11]. Monopartite begomoviruses often associate with a betasatellite to form a virus/satellite complex [15, 16]. Betasatellites are critical for the development of symptoms during the infection [17–19], and depend on the helper virus for encapsidation, movement, replication, and transmission to new host plants [20]. Interestingly, some monopartite begomoviruses from the Old World have not been found associated with betasatellites; this is the case of tomato yellow leaf curl virus (TYLCV) and tomato leaf curl Yunnan virus (TLCYnV), which can induce severe symptoms through the action of virus-encoded proteins [21–23].

The methylation of the geminiviral genome is closely connected with the plant methyl cycle [24], which provides the methyl donor, S-adenosyl methionine (SAM), for the DNA transmethylation reactions [25]. In plants, DNA methylation occurs at CG, CHG and CHH (where H is A, C or T) sequence contexts [26]. DNA methyltransferases are responsible for the addition of the methyl group on specific DNA cytosine sites, and are divided into three classes: the METHYLTRANSFERASE 1 (MET1) class is most similar to the mammalian Dnmt1 in both sequence and function, and is the major CG maintenance methyltransferase in plants [27–29]; the CHROMOMETHYLASE 3 (CMT3) class is specific to the plant kingdom, contains a chromo domain, and functions to control the maintenance of most non-CG methylation [30, 31]; the DOMAINS REARRANGED METHYLASE (DRM) class includes DRM1 and DRM2, and shows sequence similarity to the mammalian Dnmt3 methyltransferase [32]. DRM proteins are required for the initial establishment of cytosine methylation in all sequence contexts [33]; additionally, for the maintenance of CHG methylation at some loci, DRM1/2 seems to play a more important role than CMT3 [34, 35]. Methylation in asymmetric sites must be reestablished after each DNA replication cycle, because there is no complementary sequence to serve as a guide for remethylation. Since geminiviruses utilize the host DNA replication machinery to replicate the viral DNA genome, DRM-related viral DNA methylation establishment and maintenance may exert important functions in TGS of the viral genome.

TGS plays an important role in the host defense against geminiviruses, since methylation of the viral genome impairs the transcription of viral genes [24, 36]. As a counter-defense strategy, geminiviruses encode proteins that serve as suppressors of TGS, hence promoting viral replication and spread. Suppressors of TGS encoded by geminiviruses exert their functions through divergent mechanisms. Well-known geminiviral TGS suppressors are the AC2 or AL2 protein encoded by some bipartite begomoviruses, such as cabbage leaf curl virus (CaLCuV) and TGMV, and the C2 or L2 protein encoded by some curtoviruses, such as beet curly top virus (BCTV) and beet severe curly top virus (BSCTV). Both AC2/AL2 and C2/L2 suppress TGS by interfering with the methyl cycle through inactivation of adenosine kinase (ADK) [37–39]. Additionally, BSCTV C2 attenuates 26S proteasomal degradation of S-adenosyl-methionine decarboxylase (SAMDC1) [40]. TGMV-encoded AC2 was shown to interact with the H3K9 histone methyltransferase SUVH4/KYP to suppress its activity to attenuate TGS [41]. The βC1 protein encoded by the TYLCCNV-associated betasatellite blocks the methyl cycle through the interaction with the key enzyme S-adenosyl homocysteine hydrolase (SAHH) [42]. Furthermore, the replication-associated proteins (Rep, also known as C1, AC1, or AL1) of several geminiviruses were also reported to act as TGS suppressors, since they reduce the expression of the maintenance methyltransferases MET1 and CMT3 [36]. The V2 protein encoded by cotton leaf curl Multan virus (CLCuMuV) acts as a TGS suppressor through its interaction with AGO4 [43]. Finally, the C4 or AC4 protein encoded by some geminiviruses has also been described to act as TGS suppressor [21, 44]. The C4 protein from CLCuMuV interacts with S-adenosyl methionine synthetase (SAMS) and inhibits its enzymatic activity [44]; whether this is the mechanism underpinning the TGS suppression mediated by other geminivirus-encoded C4 proteins remains to be determined.

TLCYnV is a recombinant virus with TYLCCNV as major parent and pepper yellow leaf curl China virus (PepYLCCNV) as the donor of the *C4* gene and a partial intergenic region (IR). The C4 protein encoded by TLCYnV is not only a pathogenicity determinant but also suppresses methylation-mediated TGS [21–23]. In this study, we report that TLCYnV C4 interacts with NbDRM2, a major plant DNA methyltransferase. TLCYnV C4 impairs the DNA-binding ability of NbDRM2 through direct protein-protein interaction. Silencing *NbDRM2* suppresses methylation of the TLCYnV genome and enhances the viral infection in *Nicotiana benthamiana* plants. Our results provide additional evidence of the requirement of a functional methyl cycle for TGS-based anti-geminiviral defense in plants, and demonstrate that TLCYnV C4 suppresses TGS through a novel mechanism that involves the inhibition of the DNA-binding ability of NbDRM2.

## Results

### TLCYnV C4 interacts with NbDRM2

**Fig 1:**
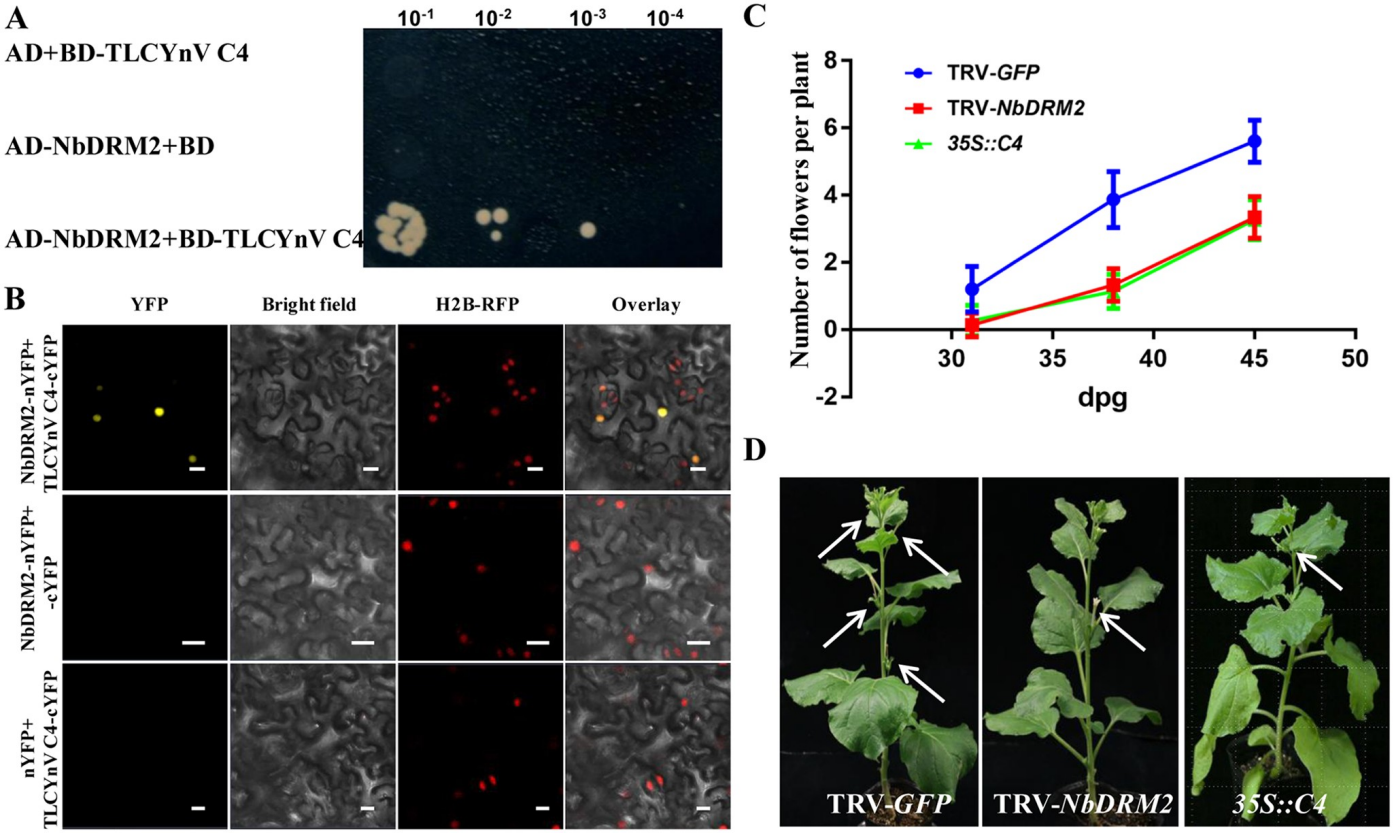
NbDRM2 interacts with TLCYnV C4. **(A)** Identification of the interaction between NbDRM2 and TLCYnV C4 in yeast cells. The yeast cells (strain Gold) co-transformed with the indicated plasmids was subjected to 10-fold series dilution, and grown on a SD/-Leu/-Trp/-His/-Ade medium. **(B)** BiFC visualization of the interaction between NbDRM2 and TLCYnV C4 in H2B-RFP transgenic *Nicotiana benthamiana* leaves. The combinations of NbDRM2-nYFP/cYFP and nYFP/TLCYnV C4-cYFP serve as negative controls. Expression of H2B was used a nuclear marker (red). This experiment was repeated three times with the similar results. Scale bar = 50 μm; **(C)** *NbDRM2*-silenced *N*. *benthamiana* plants phenocopy *35S*::*TLCYnV C4* transgenic *N*. *benthamiana* plants on the late flowering phenotype. Blue, red, and green curves represent number of flowers at given times among mock (TRV-*GFP*), *NbDRM2*-silenced (TRV-*NbDRM2*), and *35S*::*TLCYnV C4* transgenic (*35S*::*C4*) *N*. *benthamiana* plants. X-axis indicates days post-germination (dpg), Y-axis represents the average number of flowers per plant. Over 45 *N*. *benthamiana* plants in three independent experiments were used to count the flower numbers at 31, 38, and 45 dpg. The error bars are ±SD of the mean. **(D)** Phenotype of mock (TRV-*GFP*), *NbDRM2*-silenced (TRV-*NbDRM2*) and *35S*::*TLCYnV C4* transgenic (*35S*::*C4*) *N*. *benthamiana* plants. Arrows indicate flowers. Photographs were taken at 35 dpg.

To elucidate the molecular mechanism by which TLCYnV C4 suppresses TGS, we screened a *N*. *benthamiana* cDNA library by using yeast two-hybrid (Y2H) to identify C4-interacting proteins. Among the C4-interacting factors, one cDNA encoding a DNA methyltransferase was recovered. Interestingly, this protein is a novel interactor of geminivirus C4. We named this gene *NbDRM2*, since its sequence shares high similarity with that of *NtDRM2* [45]. We validated the interaction between TLCYnV C4 and NbDRM2 in Y2H and bimolecular fluorescence complementation (BiFC) assays (Fig 1A and 1B). NbDRM2 has orthologues in solanaceous plant species (NaDRM2, NtDRM2, StDRM2, and CaDRM2) (S1A Fig). A computational analysis by using SMART (SMART, http://smart.embl-reidelbery.de) predicted NbDRM2 to harbor a conserved catalytic DNA-cytosine methyltransferase domain (483–597 aa) (S1B Fig), which shares high similarity with that of orthologues (S1C Fig). To identify whether the TLCYnV C4/NbDRM2 interaction identified above has biological relevance, we silenced *NbDRM2* in *N*. *benthamiana* by using virus-induced gene silencing (VIGS) with a tobacco rattle virus (TRV)-based vector. Real-time quantitative reverse transcription PCR (qRT-PCR) analysis revealed that the transcripts of *NbDRM2* were significantly reduced (over 50%) in *NbDRM2*-silenced *N*. *benthamiana* plants when compared with those in *N*. *benthamiana* plants inoculated with the control (TRV-*GFP*) (S2 Fig). Interestingly, *NbDRM2*-silenced *N*. *benthamiana* plants showed a late-flowering phenotype compared with mock (TRV-*GFP*) *N*. *benthamiana* plants, and phenocopied *35S*::*TLCYnV C4* transgenic *N*. *benthamiana* plants (Fig 1C and 1D). These results suggest that TLCYnV C4 might interfere with the physiological functions of NbDRM2 through protein-protein interaction.

### NbDRM2-mediated methylation of the viral genome plays an important role in the plant defense against TLCYnV

**Fig 2:**
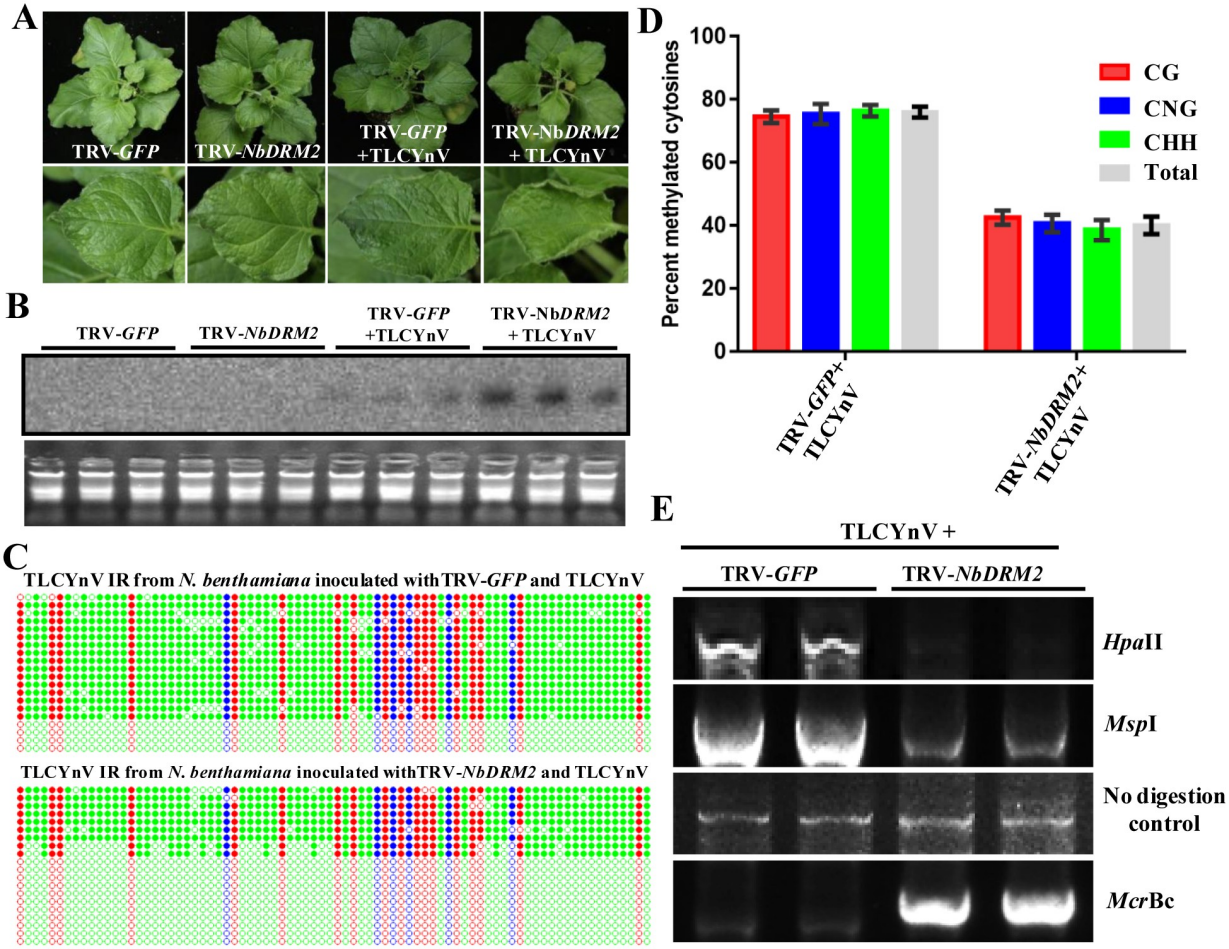
NbDRM2 plays a critical role in the defense against TLCYnV through methylating the viral genome. **(A)** *NbDRM2*-silenced *N*. *benthamiana* plants showed enhanced symptoms upon TLCYnV infection when compared with mock plants. Photographs were taken at 14 days post-inoculation (dpi). **(B)** Southern blot analysis of the TLCYnV accumulation in mock and *NbDRM2*-silenced *N*. *benthamiana* plants. Total nucleic acids (15 μg) were extracted at 14 dpi. The blot was hybridized with a *TLCYnV CP* probe. Total genomic DNA visualized by ethidium bromide staining is shown below as loading control. **(C)** Cytosine methylation profiles assessed by bisulfite sequencing. The circles represent cytosine residues and are color coded according to the sequence context (red for CG, blue for CHG, and green for CHH). Solid circles indicate methylated cytosines. Each line represents the sequence of an individual clone. **(D)** Percentage of methylated cytosines in the TLCYnV intergenic region sequences. Samples were prepared by pooling nine leaves from nine systemically infected plants at 14 dpi. Error bars represent ±SD of the mean. **(E)** Analysis of DNA methylation of the TLCYnV genome by methylation-sensitive PCR. The full-length viral genome was amplified for the methylation-sensitive PCR. Genomic DNA was digested with *Hap*II, *Msp*I, or *Mcr*Bc and then used as template for PCR. Undigested genomic DNA was used as control.

To elucidate whether NbDRM2 has biological relevance in the virus infection, we inoculated *NbDRM2*-silenced (TRV-*NbDRM2*) and mock (TRV-*GFP*) *N*. *benthamiana* plants with TLCYnV. The symptoms of the viral infection in *NbDRM2*-silenced plants were more severe than those of mock plants, and the mean latent period appeared reduced (Fig 2A and S3 Fig). As expected, Southern blot analysis showed that TLCYnV accumulation is higher in *NbDRM2*-silenced *N*. *benthamiana* plants than in mock (TRV-*GFP*) plants (Fig 2B). To test whether NbDRM2 plays an important role in the methylation of the TLCYnV genome, bisulfite sequencing was performed to detect the methylation of the viral intergenic region (IR) at high resolution. The TLCYnV IR contains 18 CG, 6 CHG, and 56 CHH sites. The bisulfite sequencing results indicate that the methylation level of the TLCYnV IR is significantly lower in *NbDRM2*-silenced *N*. *benthamiana* plants than in mock plants (Fig 2C and 2D). To confirm the contribution of NbDRM2 to the methylation of the TLCYnV genome, methylation-sensitive PCR was performed to examine the methylation status of the viral DNA in TLCYnV-infected mock and *NbDRM2*-silenced *N*. *benthamiana* plants. *Hpa*I, and *Msp*I are methylation-sensitive endonucleases whose cleavage activities are blocked by methylation of cytosine in their target sites; *Mcr*Bc is a methylation-dependent endonuclease that preferentially digests methylated DNA. Total genomic DNA was extracted and digested with those three enzymes; the PCR product was significantly decreased in *Hpa*II- and *Msp*I-digested samples from TLCYnV-infected *NbDRM2*-silenced (TRV-*NbDRM2*) *N*. *benthamiana* plants, compared to samples from TLCYnV-infected mock (TRV-*GFP*) plants, while the PCR products were significantly increased in *Mcr*Bc-digested samples (Fig 2E). These results suggest that NbDRM2 plays a critical role in the methylation of the TLCYnV genome.

### The interaction between TLCYnV C4 and NbDRM2 alters the nuclear distribution pattern of NbDRM2

**Fig 3:**
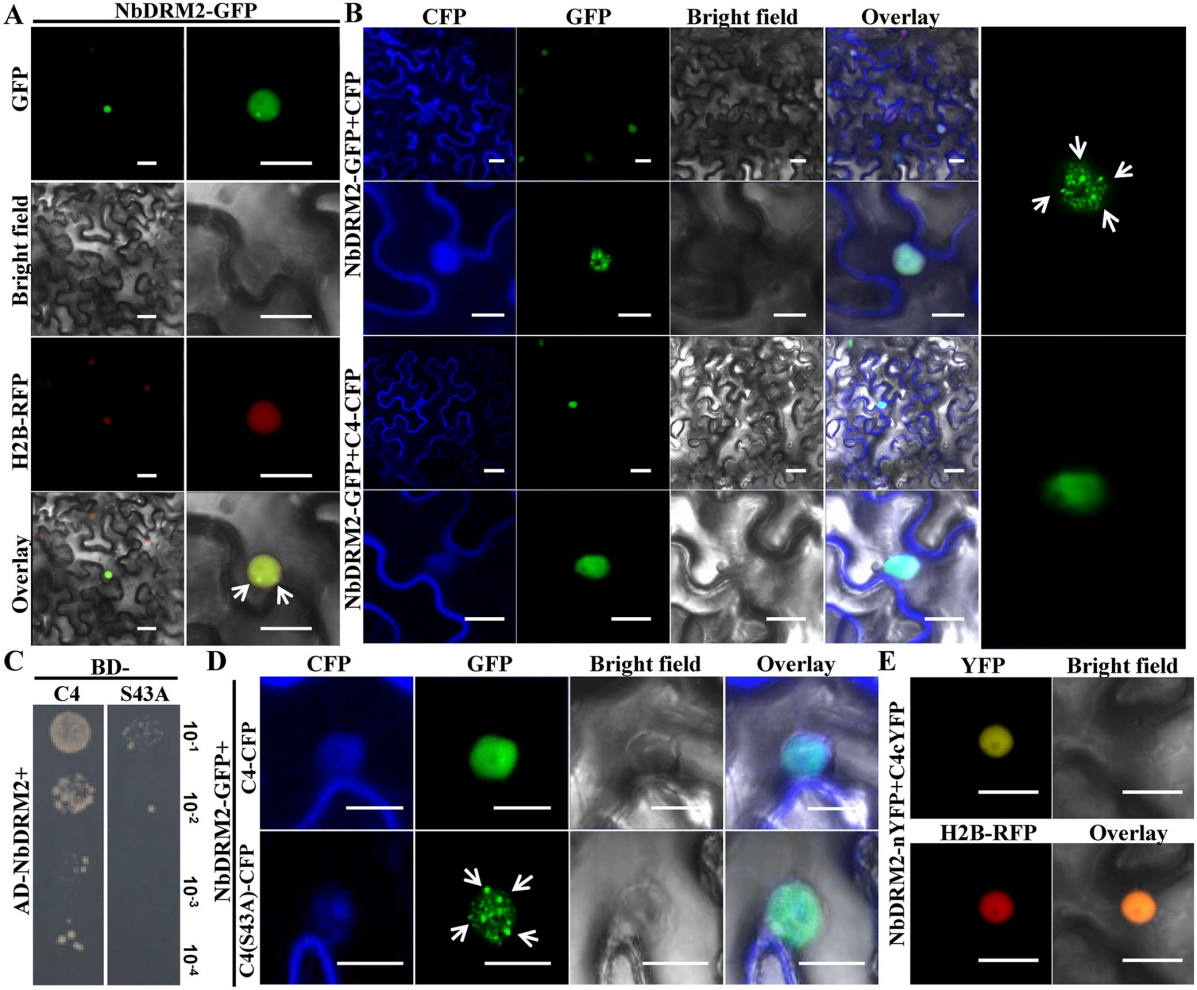
TLCYnV C4 alters the nuclear distribution pattern of NbDRM2 through the TLCYnV C4/NbDRM2 interaction. **(A)** Nuclear distribution pattern of NbDRM2-GFP transiently expressed in epidermal cells of H2B-RFP transgenic *N*. *benthamiana* plants. Arrows indicate NbDRM2 accumulation speckles in the nucleus. Scale bar = 50 μm. **(B)** NbDRM2 nuclear distribution pattern in the presence of CFP or TLCYnV C4-CFP. Arrows indicate NbDRM2 accumulation speckles in the nucleus. Scale bar = 50 μm. The right panels represent the NbDRM2-GFP nuclear distribution in the higher magnitude micrographs in the presence of CFP or TLCYnV C4-CFP. **(C)** Identification of the key site of TLCYnV C4 for the C4/NbDRM2 interaction in Y2H assays. The yeast strain Gold co-transformed with the indicated plasmids was subjected to 10-fold series dilution, and grown on a SD/-Leu/-Trp/-His/-Ade medium. **(D)** Nuclear distribution patterns of NbDRM2 in presence of TLCYnV C4 or C4 (S43A). Arrows indicate NbDRM2 accumulation speckles in the nucleus. Scale bar = 50 μm. **(E)** Detection of the nuclear distribution of the TLCYnV C4/NbDRM2 complex in BiFC assays. Scale bar = 50 μm.

To investigate the subcellular localization of NbDRM2, a construct to express NbDRM2-GFP (where the GFP protein is fused to the C-terminus of NbDRM2) from the cauliflower mosaic virus (CaMV) *35S* promoter was infiltrated into H2B-RFP transgenic *N*. *benthamiana* plants. Confocal imaging showed that NbDRM2-GFP localizes in the nucleus and forms speckles (Fig 3A). This specific nuclear localization of NbDRM2-GFP raised the possibility that NbDRM2 might bind chromosomal DNA. To further examine the localization of NbDRM2-GFP in the nucleus, we isolated intact nuclei and observed them under the confocal microscope after staining them with propidium iodide (PI). Images show that NbDRM2-GFP co-localized with the PI signal, especially in the observed speckles (S4A Fig); GFP, on the contrary, distributes evenly in the nucleus and does not form speckles (S4A Fig). These results suggest that NbDRM2 indeed binds to the plant chromosomal DNA. To investigate the influence of TLCYnV C4 on the nuclear distribution pattern of NbDRM2, we co-expressed NbDRM2-GFP with CFP or TLCYnV C4-CFP in *N*. *benthamiana*. GFP and CFP fluorescence was observed by confocal microscopy at 48 hours post-infiltration (hpi). Strikingly, the nuclear NbDRM2-GFP-containing speckles disappeared in the presence of TLCYnV C4 (Fig 3B). We also isolated intact nuclei from wild type (WT) or *35S*::*TLCYnV C4* transgenic *N*. *benthamiana* plant leaves transiently expressing NbDRM2-GFP, and observed the nuclei after PI staining, confirming that the speckles formed by NbDRM2-GFP disappeared in the presence of C4 (S4B Fig). C4 expression was demonstrated by western blot in *35S*::*TLCYnV C4* transgenic *N*. *benthamiana* plant leaves (S4C Fig). To test whether it is the interaction between TLCYnV C4 and NbDRM2 that alters the nuclear distribution pattern of NbDRM2, we aimed to identify mutations rendering C4 unable to bind NbDRM2. For this purpose, we used C4 mutants constructed in previous studies [22, 23, 46] or replaced the amino acids harboring hydroxyl groups (such as Ser or Thr) with alanine and conducted Y2H assays with NbDRM2. Our results identified Ser43 in TLCYnV C4 as required for the TLCYnV C4/NbDRM2 interaction (Fig 3C and S5 Fig). To further confirm that Ser43 of TLCYnV C4 is critical for TLCYnV C4/NbDRM2 interaction, we transiently co-expressed Flag-NbDRM2 with GFP, TLCYnV C4-GFP, or TLCYnV C4(S43A)-GFP in leaves of *N*. *benthamiana* plants and performed co-immunoprecipitation (Co-IP) assays. The Co-IP results show that the C4(S43A) mutant loses the ability to interact with NbDRM2 (S6 Fig). These results suggest that Ser43 is indeed a key site for the TLCYnV C4/NbDRM2 interaction. Interestingly, the TLCYnV C4(S43A) mutant could not alter the nuclear distribution pattern of NbDRM2 in epidermal cells of *N*. *benthamiana* plants, in sharp contrast to WT TLCYnV C4 (Fig 3D), indicating that the interaction between TLCYnV C4 and NbDRM2 is required for C4 to alter the nuclear distribution pattern of NbDRM2. To further confirm the subcellular localization of the TLCYnV C4/NbDRM2 interaction, we performed BiFC assays in the epidermal cells of H2B-RFP transgenic *N*. *benthamiana* plants. Micrographs show that the TLCYnV C4/NbDRM2 complex localizes in the nucleus and does not form speckles. These results suggest that TLCYnV C4 interferes with the DNA-binding ability of NbDRM2 to inhibit the formation of NbDRM2/DNA complex (Fig 3E). Previous studies reported that the TLCYnV C4/NbSKη interaction is critical for C4 to cause symptom-like developmental abnormalities [22, 23]. We evaluated the ability of the NbDRM2-interaction-compromised C4 mutant version to cause these symptom-like abnormalities by using a cucumber mosaic virus (CMV)-based expression vector. Mutation in Ser43 did not affect the ability of C4 to cause developmental abnormalities, ruling out a role of the TLCYnV C4/NbDRM2 in this process (S7 Fig). In agreement with this, and as expected, Ser43 of TLCYnV C4 was not a key site for the TLCYnV C4/NbSKη interaction (S8 Fig). Taken together, these results indicate that the interaction between TLCYnV C4 and NbDRM2 influences the nuclear distribution pattern of NbDRM2 but does not affect the symptom determinant activity of TLCYnV C4.

### TLCYnV C4 impacts the DNA-binding ability of NbDRM2

**Fig 4:**
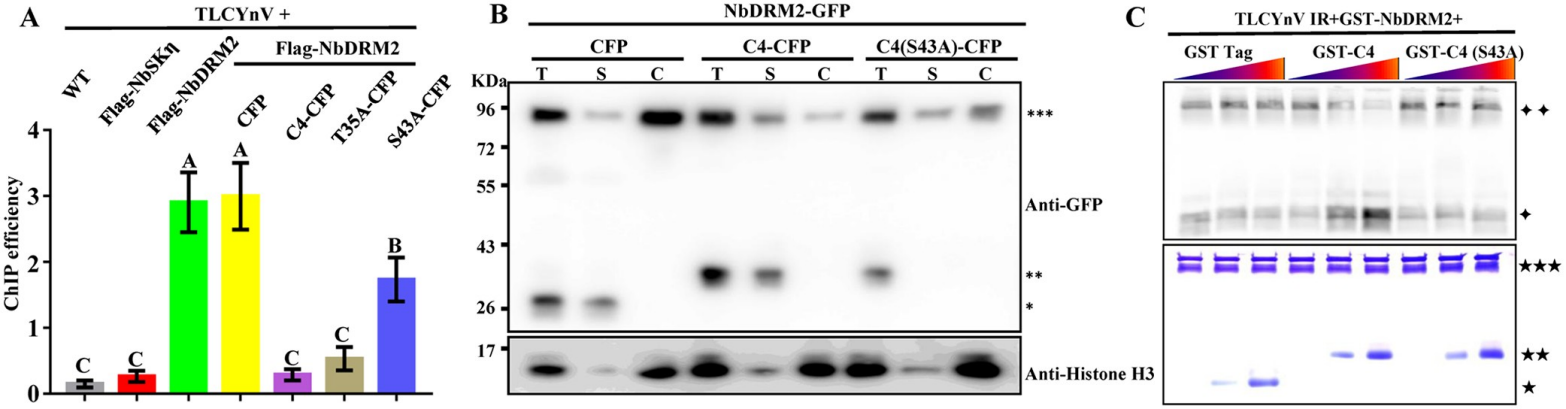
The interaction between TLCYnV C4 and NbDRM2 impairs the DNA-binding ability of NbDRM2. **(A)** ChIP-qPCR analysis of the influence of the TLCYnV C4/NbDRM2 interaction on the DNA-binding ability of NbDRM2. Systemic leaves of TLCYnV-infected *N*. *benthamiana* plants co-expressing Flag-NbDRM2 with CFP, C4-CFP, C4(T35A)-CFP, or C4(S43A)-CFP were harvested for ChIP at 48 hpi. Y-axis represents the Flag-NbDRM2 occupancy at TLCYnV intergenic region (IR). Immunoprecipitation was performed by using anti-Flag M2 magnetic beads. Immunoprecipitated DNA was eluted after reverse cross-linking and qPCR data was normalized to 1% of the input. Error bars represent ±SD of the mean. **(B)** Nuclear fraction isolation analysis of the distribution of NbDRM2 in the presence of TLCYnV C4 or the C4 (S43A) mutant. GFP was used as a marker of the soluble nucleoplasmic fraction. Histone H3 was selected as the insoluble chromatin-bound marker. T represents total nuclear extract; S and C indicate soluble and chromatin-bound (insoluble) fractions, respectively. *** indicates NbDRM2-GFP; ** indicates C4 and C4(S43A)-CFP; * indicates CFP. **(C)** Competitive EMSA analysis of the ability of NbDRM2 to bind TLCYnV IR in the presence or absence of C4. Coomassie brilliant blue staining was used as the loading control. Error bars represent ±SD of the mean. Different letters (A, B and C) denote significant differences (*P* < 0.05 determined by Tukey’s post hoc test). **✦✦** indicates NbDRM2-bound DNA; **✦** indicates free DNA. ★★★ represents GST-NbDRM2; ★★ represents GST-C4 or -C4(S43A); ★ represents GST Tag.

The altered nuclear distribution pattern of NbDRM2 caused by the TLCYnV C4/NbDRM2 association raised the possibility that TLCYnV C4 impacts the NbDRM2 DNA-binding ability through direct interaction. To test this hypothesis, chromatin immunoprecipitation-quantitative PCR (ChIP-qPCR) was performed. For this purpose, we transiently co-expressed Flag-NbDRM2 with CFP, TLCYnV C4-CFP, and C4(S43A)-CFP in systemic leaves of TLCYnV-infected *N*. *benthamiana* plants, then detected the amount of TLCYnV genomic DNA bound by NbDRM2. Interestingly, we found that the amount of TLCYnV associated to NbDRM2 was significantly decreased in the presence of TLCYnV C4, but not of the NbDRM2-interaction-compromised C4 mutant (Fig 4A and S9 Fig). To further validate that TLCYnV C4 influences the DNA-binding ability of NbDRM2, we also examined the distribution of NbDRM2 in different nuclear fractions. The transcription machinery known to be tightly associated with chromatin appears in the insoluble fraction during nuclei preparation [47]. We found that NbDRM2 was mostly detected in the insoluble fraction, with a smaller amount of protein detectable in the soluble fraction (Fig 4B). However, the amount of NbDRM2 in the insoluble fraction diminished in the presence of TLCYnV C4, with most NbDRM2 now appearing in the soluble fraction. Consistent with the histone H3 chromatin-bound nature, most of histone H3 was detected in the insoluble fraction (Fig 4B). Next, we performed a competitive electrophoretic mobility shift assay (EMSA) to identify the effect of the TLCYnV C4/NbDRM2 interaction on the DNA-binding ability of NbDRM2 *in vitro*. With this aim, we expressed GST-NbDRM2, GST-TLCYnV C4, GST-TLCYnVC4(S43A), and GST alone in *Escherichia coli* and purified them, and amplified the TLCYnV IR DNA using Alexa Fluor 680-labeled oligonucleotides. The mixtures containing GST-NbDRM2 and A860-labeled TLCYnV IR were combined with different volumes of GST Tag, GST-C4, or GST-C4(S43A). As shown in Fig 4C, more free TLCYnV IR could be detected in the mixture containing GST-TLCYnV C4 (Fig 4C). As expected, GST Tag and GST-TLCYnV C4(S43A) did not influence the amount of NbDRM2-bound TLCYnV IR DNA. These results suggest that the interaction between TLCYnV C4 and NbDRM2 indeed interferes with the DNA-binding ability of NbDRM2 and inhibits the formation of the NbDRM2/DNA complex.

### TLCYnV C4 decreases the methylation level of the viral genome through interacting with NbDRM2

**Fig 5:**
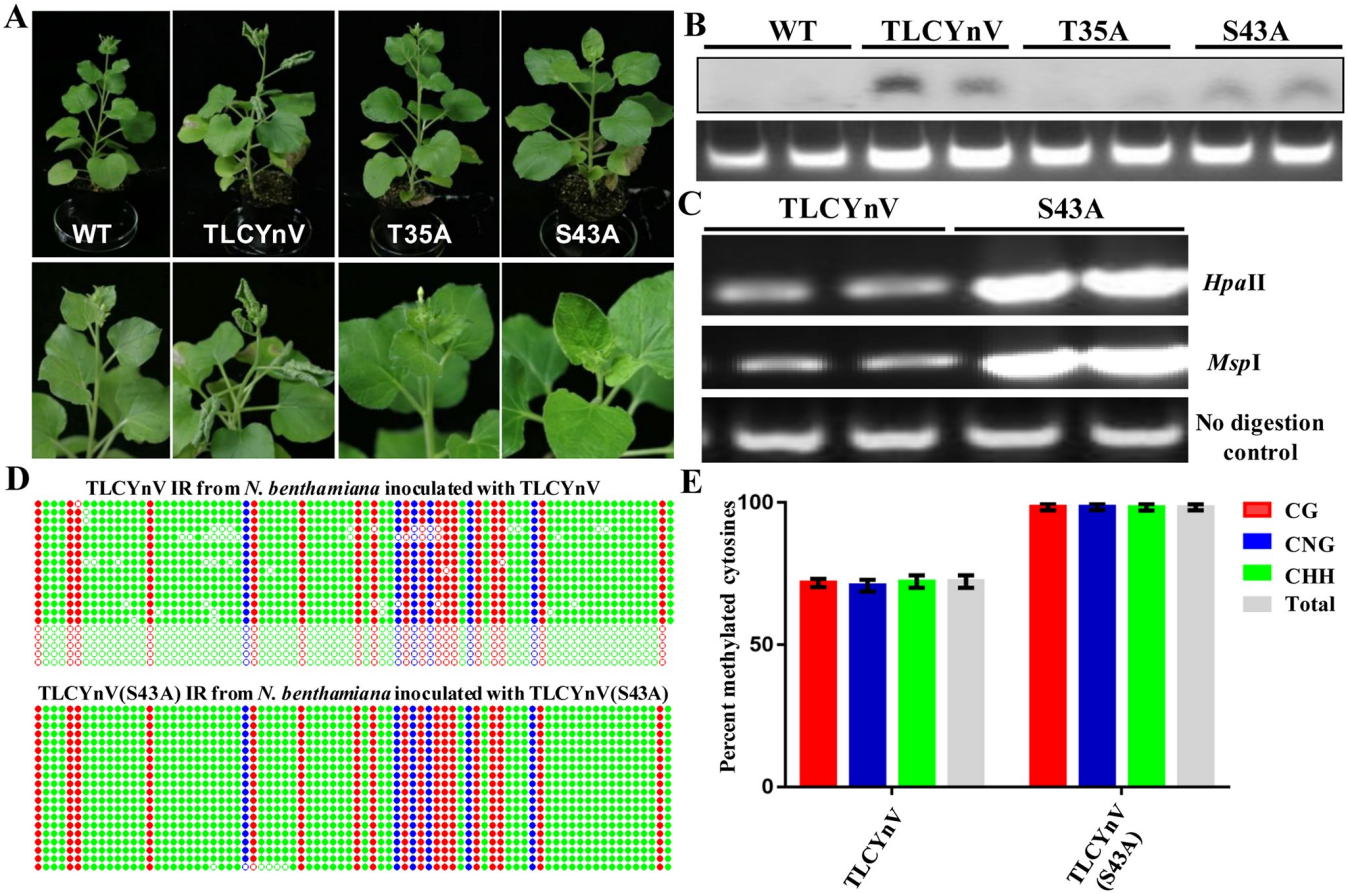
The interaction between TLCYnV C4 and NbDRM2 is critical for the viral infection. **(A)** Symptoms in *N*. *benthamiana* plants infected with TLCYnV or TLCYnV mutants encoding the NbDRM2-interaction compromised C4. Photographs were taken at 14 days post-inoculation (dpi). **(B)** Southern blot analysis of virus accumulation in systemic leaves of *N*. *benthamiana* plants infected with TLCYnV or TLCYnV mutants encoding the NbDRM2-interaction compromised C4. Total nucleic acids (15 μg) were extracted at 14 dpi. The blot was hybridized with a *TLCYnV CP* probe. Total genomic DNA visualized by ethidium bromide staining is shown below as loading control. **(C)** Methylation-sensitive PCR analysis of the methylation level of TLCYnV and TLCYnV mutant. The viral genome was amplified for the methylation-sensitive PCR. Genomic DNA was digested with *Hpa*II, or *Msp*I and then used as template for PCR. Undigested DNA was used as loading control. **(D)** Cytosine methylation profiles assessed by bisulfite sequencing. Circles represent cytosine residues and are color coded according to sequence context (red for CG, blue for CHG, and green for CHH). Solid circles indicate methylated cytosines. Each line represents the sequence of an individual clone. **(E)** Percentage of methylated cytosines in the intergenic region of TLCYnV or TLCYnV mutants encoding the NbDRM2-interaction compromised C4. Samples were prepared by pooling leaves from ten systemically infected plants at 14 dpi. Error bars represent ±SD of the mean.

To identify whether the interaction between TLCYnV C4 and NbDRM2 has biological relevance, we constructed TLCYnV infectious clones expressing WT C4, the NbSKη-interaction compromised C4 mutant [C4(T35A)], or the NbDRM2-interaction compromised C4 mutant [C4(S43A)]. Consistent with our previous results, TLCYnV expressing C4(T35A) did not infect *N*. *benthamiana* plants systemically [22], while TLCYnV expressing the C4(S43A) induced milder symptoms compared to WT TLCYnV (Fig 5A), which correlated with lower viral DNA accumulation (Fig 5B). We then performed methylation-sensitive PCR to detect the methylation level of TLCYnV and TLCYnV harboring the C4(S43A) mutant. As expected, the methylation level of the TLCYnV mutant was significantly higher than that of WT TLCYnV (Fig 5C). These results were confirmed by bisulfite sequencing (Fig 5D and 5E), demonstrating that TLCYnV C4 decreases methylation of the viral genome through the TLCYnV C4/NbDRM2 interaction. To assess the influence of this interaction on the methylation of endogenous plant DNA, we expressed TLCYnV C4, C4(T35A) and C4(S43A) mutants in *N*. *tabacum* by using a CMV-based expression vector. Consistent with previous results, expression of TLCYnV C4 (T35A) did not induce severe viral symptoms (S10A and S10B Fig). *NtGRS1*.*3*, a highly methylated repetitive DNA sequence in *N*. *tabacum* [48], was selected as a proxy to detect the influence of the TLCYnV C4/DRM2 interaction on endogenous gene methylation. We extracted total DNA and detected the methylation level of *NtGRS1*.*3* by using a methylation-sensitive restriction enzyme (*Msp*I). Southern blot analysis showed that more unmethylated *NtGRS1*.*3* could be detected in the presence of TLCYnV C4 and C4(T35A), but not of C4(S43A) (S10C Fig). To further confirm the above results, we performed methylation-sensitive PCR to detect the methylation level of *NtGRS1*.*3*; the results show that the TLCYnV C4/DRM2 interaction indeed decreases the methylation level of *NtGRS1*.*3* in *N*. *tabacum* plants (S10D Fig). Taken together, these results suggest that TLCYnV C4 decreases the methylation level of both viral and host plant genomes through interacting with NbDRM2.

### The interaction between TLCYnV C4 and NbDRM2 contributes to suppress TGS

**Fig 6:**
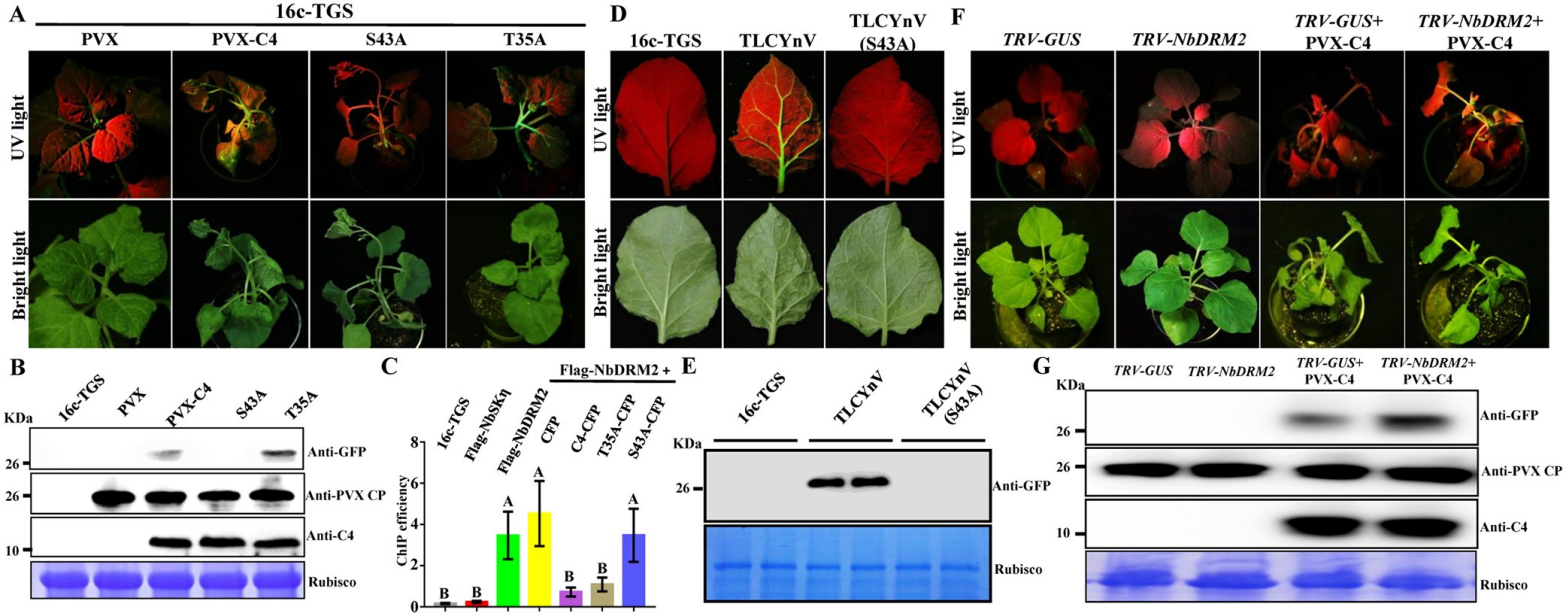
TLCYnV C4 reverses TGS through the interaction with NbDRM2. **(A)** 16c-TGS transgenic *N*. *benthamiana* plants were inoculated with PVX, PVX-C4, PVX-C4(T35A), and PVX-C4(S43A), and photographed under UV light at 12 days post-inoculation (dpi). **(B)** Western blot analysis of GFP accumulation in infected 16c-TGS transgenic *N*. *benthamiana* plants using the antibodies specific to the indicated proteins. **(C)** ChIP qPCR analysis of influence of TLCYnV C4 on CaMV *35S* promoter-binding ability of NbDRM2. Leaves of 16c-TGS *N*. *benthamiana* plants co-expressing Flag-NbDRM2 with CFP, C4-CFP, C4(T35A)-CFP, or C4(S43A)-CFP were harvested for ChIP at 48 hpi. Y-axis represents the Flag-NbDRM2 occupancy at CaMV 35S promoter. Immunoprecipitation was performed by using anti-GFP magnetic beads. Immunoprecipitated DNA was eluted after reverse cross-linking and qPCR data was normalized to 1% of the input. Error bars represent ±SD of the mean. Different letters (A and B) denote significant differences (*P* < 0.05 determined by Tukey’s post hoc test). **(D)** GFP restoration of 16c-TGS transgenic *N*. *benthamiana* plants infected by TLCYnV and TLCYnV(S43A) mutant. Photographs were taken at 12 dpi. **(E)** Western blot analysis of GFP accumulation of 16c-TGS transgenic *N*. *benthamiana* plants infected by TLCYnV WT and the TLCYnV(S43A) mutant with an anti-GFP monoclonal antibody. **(F)** TLCYnV C4 reverses TGS in *NbDRM2*-silenced 16c-TGS transgenic *N*. *benthamiana* plants at 7 dpi. **(G)** Western blot analysis of TLCYnV C4 accumulation in infected 16c-TGS transgenic *N*. *benthamiana* plants using the antibodies specific to the indicated proteins. Rubisco was used as loading control.

Given that TLCYnV C4 inhibits NbDRM2 function through protein-protein interaction, we assumed that it might suppress the NbDRM2-mediated TGS. We expressed TLCYnV C4 and C4 mutants in 16c-TGS transgenic *N*. *benthamiana* plants, in which the GFP transgene driven by the CaMV *35S* promoter is transcriptionally silenced, by using a potato virus X (PVX)-based expression vector. 16c-TGS plants infected by PVX-TLCYnV C4 or PVX-TLCYnV C4(S43A) showed abnormal development, including foliar distortion and longer internodes and petioles (Fig 6A). These phenotypes were consistent with previous reports describing TLCYnV C4 as a symptom determinant [21]. On the contrary, PVX-TLCYnV C4(T35A)-infected 16c-TGS plants only showed PVX-like symptoms, with mild chlorotic spots on leaves, consistent with our previous observations [22, 23]. Interestingly, although the NbSKη interaction-compromised TLCYnV C4 mutant [C4(T35A)] failed to induce infection-like symptoms, we found that PVX-TLCYnV C4(T35A) could suppress TGS in 16c-TGS transgenic *N*. *benthamiana* plants (Fig 6A), while the TLCYnV C4 mutant deficient in NbDRM2-binding could not (Fig 6A); this visual evaluation was confirmed by western blot to detect GFP (Fig 6B). To identify whether the *35S* promoter-binding ability of NbDRM2 is altered in the presence of TLCYnV C4 in 16c-TGS transgenic *N*. *benthamiana* plants, we transiently co-expressed NbDRM2 with CFP, C4-CFP, C4(T35A)-CFP, or C4(S43A)-CFP in 16c-TGS transgenic *N*. *benthamiana* plants and ChIP-qPCR assays were conducted. Our results show that the interaction between TLCYnV C4 and NbDRM2 indeed impairs the *35S* promoter-binding ability of NbDRM2 (Fig 6C and S11 Fig). When we inoculated 16c-TGS transgenic *N*. *benthamiana* plants with TLCYnV or the TLCYnV-C4(S43A) mutant, we found that infection by TLCYnV restores GFP expression at 12 dpi, but the TLCYnV mutant fails to do so (Fig 6D and 6E). In order to confirm an involvement of NbDRM2 in TGS of the GFP transgene in 16c-TGS plants, we silenced *NbDRM2* in these plants by using a TRV-based vector, qRT-PCR analysis shows that the transcripts of *NbDRM2* are significantly reduced (over 50%) in *NbDRM2*-silenced 16c-TGS transgenic *N*. *benthamiana* plants when compared with those in 16c-TGS transgenic *N*. *benthamiana* plants inoculated with the control (TRV-*GUS*) (S12 Fig). PVX-TLCYnV C4 was then inoculated in mock (TRV-*GUS*) and *NbDRM2*-silenced (TRV-*NbDRM2*) 16c-TGS transgenic *N*. *benthamiana* plants. As shown in Fig 6F and 6G, GFP expression was more strongly restored in *NbDRM2*-silenced 16c-TGS plants. These results suggest that the TLCYnV C4/NbDRM2 interaction plays a critical role in TGS suppression.

## Discussion

Geminiviruses replicate their genome via dsDNA intermediates in the nucleus, which could be the target of methylation-mediated TGS [49]. In this study, we report that the C4 protein encoded by TLCYnV interacts with NbDRM2, a DNA methyltransferase required for the establishment and maintenance of DNA methylation, to inhibit its physiological function and break the TGS-mediated defense.

How does the interaction between TLCYnV C4 and NbDRM2 lead to suppressed TGS? Our results show that NbDRM2 localizes in the nucleus and forms speckles, which correlates with its DNA-binding capacity. However, TLCYnV C4 can interfere with the DNA-binding ability of NbDRM2, inhibiting the formation of DNA-NbDRM2 complexes and retaining NbDRM2 in the nucleoplasm. Inhibition of NbDRM2 DNA binding by TLCYnV C4 interferes with the NbDRM2-mediated DNA methylation. This model is supported by several pieces of evidence: (1) The nuclear speckles potentially containing DNA-bound NbDRM2 disappear in the presence of TLCYnV C4; (2) A higher proportion of soluble NbDRM2 can be detected in the presence of TLCYnV C4; (3) The TLCYnV infectious clone expressing the NbDRM2-interaction compromised C4 mutant has weaker pathogenicity and higher methylation levels of the viral genome; (4) TLCYnV C4, but not the mutant deficient in NbDRM2 interaction, decreases the methylation level of a DRM2 target locus. Taken together, these results strongly suggest that TLCYnV C4 impacts NbDRM2-mediated TGS through the interaction with this protein.

Although DRM2 plays an important role in maintenance of DNA methylation, only partial GFP could be restored in the systemic leaves of *NbDRM2*-silenced 16c-TGS transgenic *N*. *benthamiana* plants (Fig 6D). Strikingly, GFP expression was more strongly restored in *NbDRM2*-silenced 16c-TGS plants infected with PVX-TLCYnV C4 (Fig 6D). These results could be explained by at least two different possibilities: (1) Other proteins that function redundantly with NbDRM2 might exist and interact with TLCYnV C4; (2) The reduction of *NbDRM2* in the silenced plants might be insufficient to restore GFP expression in 16c-TGS plants. Further efforts are necessary to explore the role of the methylation marks deposited by NbDRM2 in transcriptionally silenced GFP of 16c-TGS transgenic *N*. *benthamiana* plants.

Previous studies reported that TLCYnV C4 is the viral symptom determinant and induces symptoms through the TLCYnV C4/NbSKη interaction [21–23]. Interestingly, a residue (S43) essential for the TLCYnV C4/NbDRM2 interaction, does not affect the interaction between TLCYnV C4 and NbSKη. When we expressed the NbDRM2 interaction-compromised C4 mutant [C4(S43A)] in *N*. *benthamiana* by using a CMV-based expression vector and, the C4 mutant [C4(S43A)] produces developmental abnormalities similarly to WT C4 (S7 Fig); on the other hand, the TLCYnV C4 mutant [C4(T35A)] deficient in NbSKη interaction can suppress TGS in 16c-TGS transgenic *N*. *benthamiana* plants. Therefore, our results support the idea that the multifunctional C4 protein exerts its symptom determinant activity and its TGS-suppression ability by independent mechanisms, and that these two processes can be uncoupled.

Ser43 of TLCYnV C4 is critical for the interaction between TLCYnV C4 and NbDRM2. A C4 protein mutated in this residue [C4(S43A)] does not impact the DNA-binding ability of NbDRM2, as opposed to the wild-type C4 (Figs 4C and 6C). However, C4(S43A) could not completely inhibit the DNA-binding ability of NbDRM2, especially the viral DNA-binding ability (Fig 4A). These results suggest that another amino acid(s) might be involved in the TLCYnV C4/NbDRM2 interaction. Further efforts to investigate new site(s) important for the TLCYnV C4/NbDRM2 interaction will provide a more complete understanding of the molecular mechanism by which TLCYnV C4 impacts the DNA-binding ability of NbDRM2, and in turn TGS.

In this study, we show that TLCYnV C4 interferes with the DNA-binding ability of NbDRM2 to inhibit the NbDRM2-mediated viral genomic DNA methylation. We also found that the TLCYnV C4/NbDRM2 interaction decreased the methylation of one DRM2-targeted endogenous gene (*NtGRS1*.*3*) in *N*. *tabacum* (S10 Fig). This finding suggests that the interaction between TLCYnV C4 and DRM2 not only impacts TGS-mediated defense against geminiviruses, but has a general effect on DRM2 targets in plants; this idea is consistent with the effect of C4 on the nuclear distribution of NbDRM2 in the absence of the virus. Whether C4, through its interaction with NbDRM2, impacts genome-wide plant DNA methylation remains to be determined.

**Fig 7:**
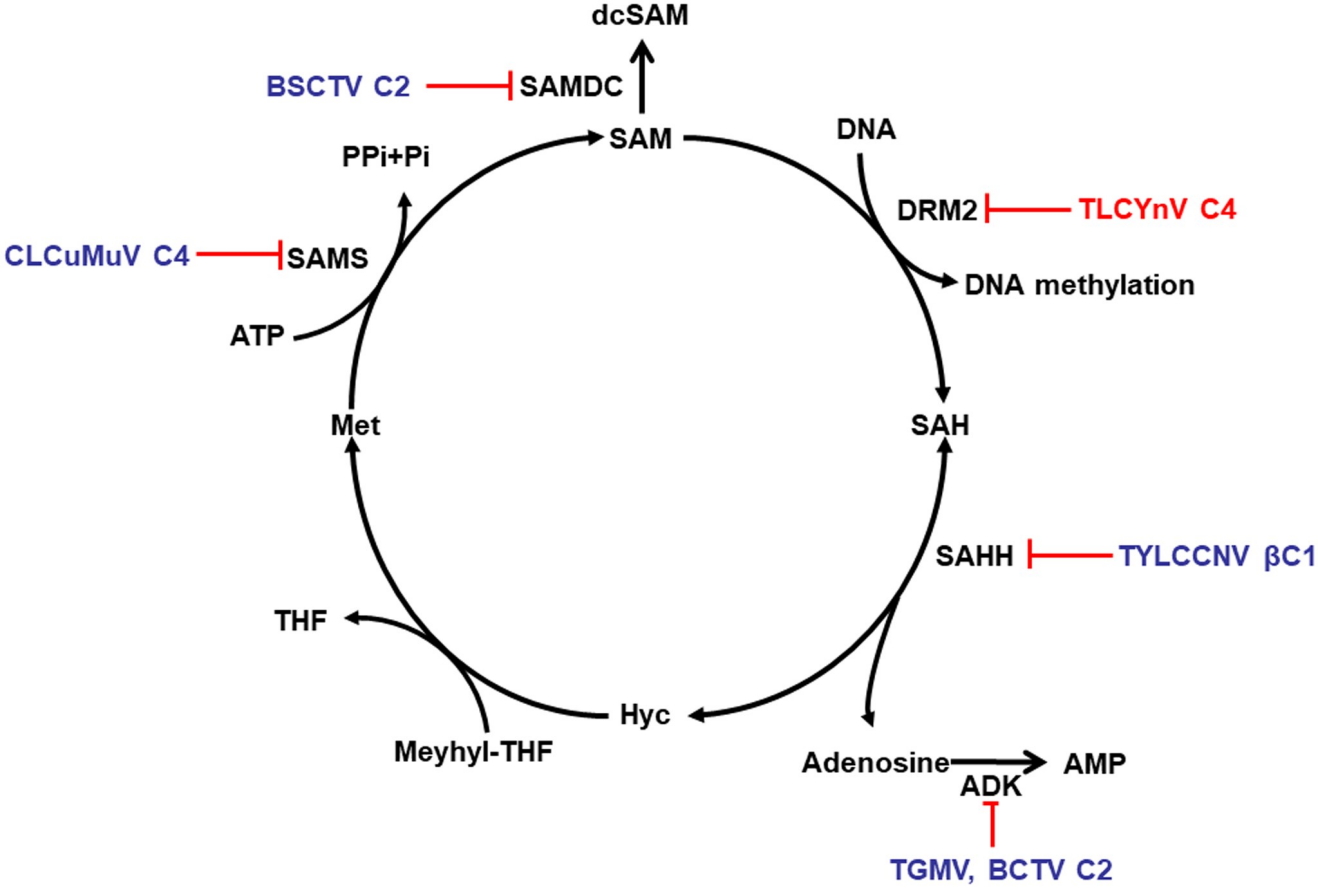
Targets of geminivirus-encoded proteins in the methyl cycle. S-adenosyl methionine (SAM) is the methyl donor for most transmethylation reactions. Its product, S-adenosyl homocysteine (SAH), inhibits transmethylation by competing with SAM for methyltransferases (MTases). SAH is converted to homocysteine (Hyc) and adenosine by S-adenosyl homocysteine hydrolase (SAHH). The phosphorylation of adenosine by adenosine kinase (ADK) is critical because the SAHH-catalyzed reaction is reversible and the equilibrium lies in the direction of SAM synthesis. By removing adenosine, ADK promotes flux through the cycle and SAM production, and minimizes competitive inhibition of the methyltransferase reaction by SAH. Geminivirus AC2/AL2 and C2/L2 proteins inactivate ADK and have also been shown to stabilize SAM decarboxylase (SAMDC), which causes decarboxylated SAM (dcSAM) levels to rise. The TYLCCNV betaC1 protein directly antagonizes the methyl cycle by inhibiting SAHH. The C4 protein encoded by CLCuMuV interacts with S-adenosyl methionine synthesase (SAMS) to inhibit SAMS activity for TGS suppression. As described in this work, TLCYnV C4 reverses TGS through interacting with and impacting the DNA-binding ability of NbDRM2.

Based on previous research and the work presented here, we propose a model to explain how TLCYnV C4 suppresses TGS. In the absence of TLCYnV C4, NbDRM2 binds to the viral DNA to silence gene expression; the presence of C4 negatively impacts the DNA-binding ability of NbDRM2 and inhibits the formation of the NbDRM2/DNA complex, consequently blocking the NbDRM2-mediated gene silencing (Fig 7). Strikingly, multiple geminiviral proteins encoded by different species have evolved to target the methyl cycle, in turn suppressing anti-viral gene silencing. For example, AC2/AL2 and C2/L2 proteins inactivate ADK and stabilize SAM decarboxylase (SAMDC), which causes decarboxylated SAM (dcSAM) levels to rise [37–39, 40]; TYLCCNV βC1 protein inhibits SAHH [42]; the C4 protein encoded by CLCuMuV interacts with and inhibits SAMS [44] (Fig 7). The convergent targeting of this pathway by geminiviruses underscores its importance in anti-geminiviral defence, and highlights its potentiality for the design of strategies to engineer resistance to the devastating crop diseases caused by these viruses.

## Materials and methods

### Plant material and growth conditions

Transgenic *N*. *benthamiana* plants expressing the nuclear marker H2B-RFP (full-length red fluorescent protein fused to the C-terminus of histone 2B) were kindly provided by Dr. Michael M. Goodin (University of Kentucky. KY, USA) [50]. Transgenic *N*. *benthamiana* plants expressing TLCYnV C4 were described previously [21]. *N*. *benthamiana* plants were grown inside a growth chamber at 26°C, under a 16-h light/8-h dark photoperiod.

### Plasmids construction

*NbDRM2* was cloned into p2YN, pGADT7, pGD-Flag, pGex4T-3, and pGD-GFP vectors, and TLCYnV *C4* was cloned into p2YC, pGBKT7, pCHF3, pCHF3-CFP, pGex4T-3 and pgR106 vectors. C4(G2A), C4(P32A), C4(P33A), C4(N34A), C4(T35A), C4(T36A), C4(T38A), C4(S39A), C4(S43A), C4(T47A), C4(S49A), C4(T51A), C4(T55A), C4(T59A), and C4(T60A) were cloned individually into pGBKT7. C4(T35A) and C4(S43A) were cloned individually into pgR106 and pCHF3-CFP vectors. The part of the coding sequence of *NbDRM2* was cloned into pTRV-RNA2 for VIGS. All cDNAs were PCR-amplified using the KOD-Plus-Neo High-Fidelity DNA polymerase (TOYOBO). The resulting PCR fragments were first cloned individually into the pMD18-T vector (TaKaRa), then released from the vector using corresponding restriction enzymes. The released fragments were ligased into the expression vectors. C4(P32A), C4(P33A), C4(N34A), C4(T35A), C4(T36A), C4(T38A), C4(S39A), C4(S43A), C4(T47A), C4(S49A), C4(T51A), C4(T55A), C4(T59A), and C4(T60A) mutants were generated by the overlapping PCR methods. All primers used for plasmids construction are listed in Supplemental Table 1 (S1 Table).

### Y2H assays

Y2H assays were performed essentially as described previously [22].

### BiFC assays

BiFC assays were conducted on 5-week-old transgenic H2B-RFP *N*. *benthamiana* leaves infiltrated with a combination of *A*. *tumefaciens* C58C1 carrying p2YN-TLCYnV C4 (to produce TLCYnV C4-nYFP) and *A*. *tumefaciens* C58C1 carrying p2YC-NbDRM2 (to produce NbDRM2-cYFP). Emission of the YFP interaction signal was detected using Zeiss LSM 780 laser scanning microscope (Carl-Zeiss) at 48 hpi.

### Co-IP assays

Co-IP assays were conducted essentially as described previously [22], with minor modifications. *N*. *benthamiana* leaves (0.5 g) transiently transformed to express the proteins of interest were harvested at 2 dpi and ground in 1 mL IP buffer (50mM Tris-HCl, 150mM NaCl, 10mM MgCl_2_, 5mM DTT, and Triton X-100 0.1%), centrifuged at 8,000 *g* at 4°C for 15 min, and the soluble proteins were immunoprecipitated with 20 μL anti-Flag M2 magnetic beads at 4°C for 2 h. Following three consecutive washes with IP buffer with 10 min incubation each at 4°C, the protein complexes were eluted in 200 μL of elution buffer (200 μg/mL 3×Flag peptide, 50 mM Tris-HCl, 150 mM NaCl, 10 mM MgCl_2_, 5 mM DTT) then centrifuged at 1,600 *g* for 5 min. The supernatant and crude extracts were subjected to SDS-PAGE/western blot analysis.

### Agroinfection assays in *N*. *benthamiana*

Agroinfection assays were conducted essentially as described previously [22,23]. The constructs whose backbone is pgR106 were transformed into *A*. *tumefaciens* GV3101 by electroporation; others were introduced into *A*. *tumefaciens* C58C1 by electroporation. The transformed bacteria cultures were grown individually until approximately OD_600_ = 0.5~0.8. The cultures were collected and re-suspended using an induction buffer [10mM MgCl_2_, 100mM MES (pH 5.7), 2mM acetosyringone] for 3 h at room temperature. The suspensions were adjusted to OD_600_ = 0.5. For co-expression experiments, the individual cultures were adjusted to OD_600_ = 0.4 and equal volumes were mixed before leaf infiltration. The suspensions were infiltrated into leaves of 4- to 6-week old *N*. *benthamiana* leaves using 1-ml needleless syringes.

### Isolation of total nuclear, nucleoplasmic, and chromatin-bound protein

Isolation of chromatin-bound protein assays were performed as described previously [51–53]. Leaves co-expressing NbDRM2 with TLCYnV C4 or C4 mutants were harvested and cross-linked (1% formaldehyde), followed by nucleus isolation using Honda buffer. Three volumes of 1% SDS (10 mM Tris, pH 7.5, 2 mM EDTA, and 1× proteinase inhibitor) were added to the nucleus pellet, which was vortexed for 1 min, followed by centrifugation at 14,000 *g* for 10 min. The supernatant containing soluble nucleoplasmic proteins was collected. The remaining pellet was resuspended in 3 volumes of 1% SDS and sonicated, followed by centrifugation at 14,000 *g* for 10 min. The supernatant containing chromatin-bound proteins was collected. Total nuclear protein was isolated by adding 3 volumes of 1% SDS directly to the nucleus pellet, followed by boiling at 95°C for 10 min. Equal volumes of each fraction were mixed with loading buffer, boiled, gel-separated, and subjected to SDS-PAGE analysis.

### Methylation-sensitive PCR

Methylation-sensitive PCR assays were performed as described previously [49]. Systemic leaves of *N*. *benthamiana* plants infected by TLCYnV at 14 dpi were harvested and prepared for total genomic DNA isolation. Genomic DNA was isolated by using DNeasy Plant Minikit (Qiagen). Then, 100 ng of genomic DNA was digested for 1 h at 37 with 2U of *Hpa*II, *Msp*I, or *Mcr*Bc in 20 μl reaction mixtures. The enzymes were heat inactivated, and 2 μl of the cleaved DNA was loading into PCR mixtures containing primers for full-length TLCYnV. PCR products of undigested genomic DNA served as control.

### Bisulfite sequencing assays

Bisulfite sequencing assays were performed essentially as described previously [42–44]. Genomic DNA was extracted from plant leaves using DNeasy Plant Minikit (Qiagen). To improve the efficiency of bisulfite treatment, DNA (1 mg) was digested with *Bam*HI, which acts outside of interest to decrease the size of DNA, followed by overnight treatment with proteinase K. Bisulfite modification was carried out using the EZ DNA Methylation Gold Kit (Zymo Research). Bisulfite-modified DNA was purified using a Zymo-Spin IC column and dissolved in 20 μl of Elution Buffer according to the manufacturer’s instructions. PCR polymerase and products were cloned into a pLB vector. Individual clones were sequenced. Primers were designed against templates are listed in S1 Table.

### ChIP quantitative-PCR

ChIP quantitative PCR was preformed essentially as described previously [54–56]. Chromatin was prepared from cross-linked material using extraction buffer, sonicated, diluted, and subjected to immunoprecipitation using anti-Flag M2 magnetic beads. The immunoprecipitation was performed at 4°C for 3 h, after which the beads were washed four times with ChIP dilution buffer and twice with 1 × TE buffer (10 min each). Immunoprecipitated DNA was eluted after reverse cross-linking by boiling at 95°C for 10 min, followed by treatment with proteinase K for 1 h at 55°C. qPCR data was normalized to 1% of the input.

### EMSA assays

EMSA assays were performed essentially as described previously [57, 58]. The recombinant GST, GST-NbDRM2, GST–TLCYnV C4, and GST-TLCYnV C4(S43A) proteins were purified from *E*. *coli* strain BL21(Codonplus). The concentration of purified proteins was measured with a NanoDrop instrument (NanoDrop Technologies, Wilmington, DE, USA). To generate Alexa Fluor 680-labeled TLCYnV IR, Alexa Fluor 680-labeled oligonucleotides corresponding to the viral sequence were mixed with the PCR mixture to amplify TLCYnV IR. GST-NbDRM2 (0.5 μg) was mixed with Alexa Fluor 680-labeled TLCYnV IR, then steadily increasing amounts (0, 0.15 μg and 0.3 μg) of GST Tag, GST-C4, or GST-C4(S43A) were added into the GST-NbDRM2/TLCYnV IR mixture containing the binding buffer (25 mM Tris-HCl [pH 7.5], 5 mM MgCl_2_, 0.2 mM EDTA, 1 mM dithiothreitol [DTT], 2.5 mM ATP). The reaction mixtures were incubated at room temperature for 45 min, resolved on a 1% Tris-borate-EDTA (pH 8.5) agarose gel, and visualized by using an Odyssey infrared imaging system in the 700-nm channel.

## Supporting information

S1 Table Primers used in plasmid construction in this study. (DOC)

S1 Fig Bioinformatics analysis of the homologues and functional domain of NbDRM2. **(A)** Phylogenetic analysis of NbDRM2 homologues from different species based on the amino acid sequences using Clustal W from MegAlign software. **(B)** Schematic representation of NbDRM2 deduced from the SMART online software. **(C)** Sequence alignment of the catalytic DNA methyltransferase domain of NbDRM2 and orthologues using Meglign software.
(TIF)

S2 Fig qRT-PCR analysis of *NbDRM2* gene expression in control (TRV-*GFP*) and *NbDRM2*-silenced *N*. *benthamiana* plants. Relative accumulation level of *NbDRM2* transcripts is normalized to the *actin* transcript. Error bars represent standard deviation of three biological replicates.
(TIF)

S3 Fig *NbDRM2*-silenced *N*. *benthamiana* plants are hypersensitive to TLCYnV infection. Red and blue curves represent the time course of TLCYnV symptom development in *NbDRM2*-silenced (TRV-*DRM2*) and mock (TRV-*GFP*) *N*. *benthamiana* plants, respectively. X-axis indicates days post-inoculation (dpi); Y-axis represents the percentage of symptomatic *N*. *benthamiana* plants. Over 60 *N*. *benthamiana* plants were used in this experiment. Error bars represent standard deviation of three biological replicates.
(TIF)

S4 Fig NbDRM2 co-localizes with chromosomal DNA in isolated nuclei. **(A)** Nuclear distribution of NbDRM2 in isolated nuclei; **(B)** TLCYnV C4 influences the NbDRM2 nuclear distribution pattern in isolated nuclei. Chromosomal DNA was stained by propidium iodide (PI). Scale bar = 20 μm. **(C)** Immunoblot analysis of TLCYnV C4 accumulation in *35S*::*TLCYnV C4* transgenic *N*. *benthamiana* plants. Two independent transgenic lines were used. Rubisco was used as loading control.
(TIF)

S5 Fig Identification of the key site(s) of TLCYnV C4 for the interaction with NbDRM2. The yeast strain Gold co-transformed with the indicated plasmids was subjected to 10-fold series dilution, and grown on a SD/-Leu/-Trp/-His medium.
(TIF)

S6 Fig Ser43 of TLCYnV C4 is a key site vital for its interaction with NbDRM2. Leaves co-expressing Flag-NbDRM2 with GFP, TLCYnV C4-, or TLCYnV C4(S43A)-GFP were harvested at 2 dpi for co-immunoprecipitation (Co-IP) assays. Immunoblot analysis was conducted with antibodies specific to detect the indicated proteins.
(TIF)

S7 Fig The interaction between TLCYnV C4 and NbDRM2 does not influence the symptom determinant activity of TLCYnV C4. **(A)** Phenotype of *N*. *benthamiana* plants expressing TLCYnV C4 or C4 mutants systemically by using a CMV-based vector. The TLCYnV C4 mutant [C4(T35 A)], which does not induce viral symptoms, is used as control. Photographs were taken at 8 days post-inoculation. **(B)** Accumulation of TLCYnV C4 or C4 mutants in leaves of *N*. *benthamiana* plants under different treatments determined by Western blot using antibodies specific to the indicated proteins.
(TIF)

S8 Fig Ser43 of TLCYnV C4 is critical for the C4/NbDRM2 interaction but does not influence the interaction between TLCYnV C4 and NbSKη. The yeast strain Gold co-transformed with the indicated plasmids was subjected to 10-fold series dilution, and grown on a SD/-Leu/-Trp/-His/-Ade medium.
(TIF)

S9 Fig Accumulations of transiently expressed proteins in systemic leaves of TLCYnV-infected *N*. *benthamiana* plants determined by Western blot. C4-, C4 mutants-GFP and Flag-NbDRM2 proteins were detected using GFP and Flag specific monoclonal antibodies. Rubisco was used as the loading control.
(TIF)

S10 Fig The interaction between TLCYnV C4 and DRM2 decreases the methylation level of endogenous DRM2-targets genes in tobacco. **(A)** Phenotype of *N*. *tabacum* plants expressing TLCYnV C4 or C4 mutants by using a CMV-based vector. Photographs were taken at 12 days post-inoculation. **(B)** Accumulation of TLCYnV C4 and C4 mutants in *N*. *tabacum* leaves under different treatments determined by Western blot using the antibodies specific to the indicated proteins. **(C)** Southern blot analysis of the methylation level of *NtGRS1*.*3*. Genomic DNAs from *N*. *tabacum* plants expressing TLCYnV C4 and C4 mutants were digested with *Msp*I, then resolved in a 1.5% agarose gel. **(D)** Detection of the methylation level of *NtGRS1*.*3* by using methylation-sensitive PCR. Genomic DNA was digested with *Msp*I and then used as template for PCR. Undigested DNA was used as the loading control.
(TIF)

S11 Fig Accumulation of transiently expressed proteins in 16c-TGS transgenic *N*. *benthamiana* leaves determined by Western blot. TLCYnV C4-, C4 mutants-GFP and Flag-NbDRM2 proteins were detected using monoclonal antibodies specific for GFP and Flag. Rubisco was used as the loading control.
(TIF)

S12 Fig qRT-PCR analysis of *NbDRM2* gene expression in control (TRV-*GUS*) and *NbDRM2*-silenced 16c-TGS transgenic *N*. *benthamiana* plants. Relative accumulation of *NbDRM2* transcripts is normalized to the *actin* transcript. Error bars represent standard deviation of three biological replicates.
(TIF)
