# Supplementary material for: The C4 protein encoded by tomato leaf curl Yunnan virus reverses transcriptional gene silencing by interacting with NbDRM2 and impairing its DNA-binding ability
Source: PLoS Pathog. 2020 Oct 1;16(10):e1008829. doi: 10.1371/journal.ppat.1008829 (PMC7529289; doi:10.1371/journal.ppat.1008829)
Supplement: S1 Table — (DOC) [file ppat.1008829.s001.doc]

| S1 Table **Primers used in study** | |
| --- | --- |
| Gene | Sequence |
| **Cloning primers** |  |
| pGBKT7-TLCTnV C4-F-EcoRI | GAATTCATGGGTCACTGCATCTCCATGTG |
| pGBKT7-TLCTnV C4-R-BamHI | GGATCCGGGCCTCTGCAGCAGCATCAT |
| pGADT7- NbDRM2-F-EcoRI | GAATTCATGCAGGACAACAATCTTTCTCCAGA |
| pGADT7- NbDRM2-R-BamHI | GGATCCTAATGTCTATGTCTGGACATTATGGAC |
| p2YC-TLCYnV C4-F-PacI | TTAATTAACATGGGTCACTGCATCTCCATGTG |
| p2YC-TLCYnV C4-R-SpeI | ACTAGTGGGCCTCTGCAGCAGCATCATTAG |
| p2YN-NbDRM2-F-AscI | TTAATTAACATGCAGGACAACAATCTTTCTCCAGA |
| p2YN-NbDRM2-R-SpeI | ACTAGTTGTCTATGTCTGGACATTATGGAC |
| TLCYnV C4(P32A)-F | ATACCTGCTCCCAATACCACC |
| TLCYnV C4(P32A)-R | GGTGGTATTGGGAGCAGGTAT |
| TLCYnV C4(P33A)-F | ATACCTCCAGCTAATACCACC |
| TLCYnV C4(P33A)-R | GGTGGTATTAGCTGGAGGTAT |
| TLCYnV C4(N34A)-F | CTCCACCCGCTACCACCCAAACTTC |
| TLCYnV C4(N34A)-R | GAAGTTTGGGTGGTAGCGGGTGGAG |
| TLCYnV C4(T35A)-F | CTCCACCCAATGCTACCCAAACTTC |
| TLCYnV C4(T35A)-R | GAAGTTTGGGTAGCATTGGGTGGAG |
| TLCYnV C4(T36A)-F | CTCCACCCAATACCGCTCAAACTTC |
| TLCYnV C4(T36A)-R | GAAGTTTGAGCGGTATTGGGTGGAG |
| TLCYnV C4(T38A)-F | AATACCACCCAAGCTTCCAGGGAGCTA |
| TLCYnV C4(T38A)-R | TAGCTCCCTGGAAGCTTGGGTGGTATT |
| TLCYnV C4(T39A)-F | ACCACCCAAACTGCTAGGGAGCTAAGT |
| TLCYnV C4(T39A)-R | ACTTAGCTCCCTAGCAGTTTGGGTGGT |
| TLCYnV C4(T43A)-F | TCCAGGGAGCTAGCTCCAGCTCGGACG |
| TLCYnV C4(T43A)-R | CGTCCGAGCTGGAGCTAGCTCCCTGGA |
| TLCYnV C4(T47A)-F | TCCAGGGAGCTAGCTCCAGCTCGGACG |
| TLCYnV C4(T47A)-R | CGTCCGAGCTGGAGCTAGCTCCCTGGA |
| TLCYnV C4(T49A)-F | GCTCGGACGTCAGCTCCTACATCAAGA |
| TLCYnV C4(T49A)-R | TCTTGATGTAGGAGCTGACGTCCGAGC |
| TLCYnV C4(T51A)-F | ACGTCAAGTCCTGCTTCAAGAAGGACG |
| TLCYnV C4(T51A)-R | CGTCCTTCTTGAAGCAGGACTTGACGT |
| TLCYnV C4(T55A)-F | ACATCAAGAAGGGCTGTGATTACATCG |
| TLCYnV C4(T55A)-R | GCATGTAATCACAGCCCTTCTTGATGT |
| TLCYnV C4(T59A)-F | ACGGTGATTACAGCTACTGGGGTAGTT |
| TLCYnV C4(T59A)-R | AACTACCCCAGTAGCTGTAATCACCGT |
| TLCYnV C4(T60A)-F | GTGATTACATCGGCTGGGGTAGTTTTC |
| TLCYnV C4(T60A)-R | GAAAACTACCCCAGCCGATGTAATCAC |
| PVX-TLCYnV C4-F-AscI | GGCGCGCCCATGGGTCACTGCATCTCCATGTG |
| PVX-TLCYnV C4-R-SalI | GTCGACTTAGGGCCTCTGCAGCATC |
| pCHF3-TLCYnV C4-CFP-F-KpnI | GGTACCATGGGTCACTGCATCTCCATGTG |
| pCHF3-TLCYnV C4-CFP-R-BamHI | GGATCCGGGCCTCTGCAGCAGCATCATTAG |
| pGex4T-3- TLCYnV C4-F-BamHI | GGATCCATGGGTCACTGCATCTCCATGTG |
| pGex4T-3- TLCYnV C4-R-SalI | GTCGACTTAGGGCCTCTGCAGCAGCATCAT |
| pGD NbDRM2-GFP -F-SalI | GTCGACATGCAGGACAACAATCTTTCTCCAGA |
| pGD-NbDRM2-GFP-R-BamHI | GGATCCTGTCTATGTCTGGACATTATGGAC |
| TRV- NbDRM2-F-BamHI | GGATCCATGCTATTGGGATTTCCAAAGAACCA |
| TRV- NbDRM2-R-XhoI | CTCGAGTACCGAGACGATAAAGAGCAACTTCAC |
| pCambia-Flag- NbDRM2 -F | GGTACCATGCAGGACAACAATCTTTCTCCAGA |
| pCambia-Flag- NbDRM2-R | GGATCCTGTCTATGTCTGGACATTATGGAC |
| pGex4T-3- NbDRM2-F-BamHI | GGATCCATGCAGGACAACAATCTTTCTCCAGA |
| pGex4T-3- NbDRM2-R-SalI | GTCGACTTATGTCTATGTCTGGACATTATGGAC |
| **Quantitative-PCR primer pairs** |  |
| qPCR-NbDRM2-F | CCAATGAGAGTTCAGAAGTTTGT |
| qPCR-NbDRM2-R | CACCAGCTTCTCACAATATCCCTG |
| qPCR-35S promoter -F | CCAGTATGGACGATTCAAGGCTTG |
| qPCR-35S promoter-R | CGAGTTCTGTTAGGTCCTCTATTTGAA |
| qPCR-Silencing efficiency-NbDRM2-F | GATTCAGATTTGGTGTTGGATGCTCT |
| qPCR-Silencing efficiency-NbDRM2-R | CACATCTCTCCATTGCTATGGAAACC |
