# Supplementary figures and images for: The C4 protein encoded by tomato leaf curl Yunnan virus reverses transcriptional gene silencing by interacting with NbDRM2 and impairing its DNA-binding ability
Source: PLoS Pathog. 2020 Oct 1;16(10):e1008829. doi: 10.1371/journal.ppat.1008829 (PMC7529289; doi:10.1371/journal.ppat.1008829)

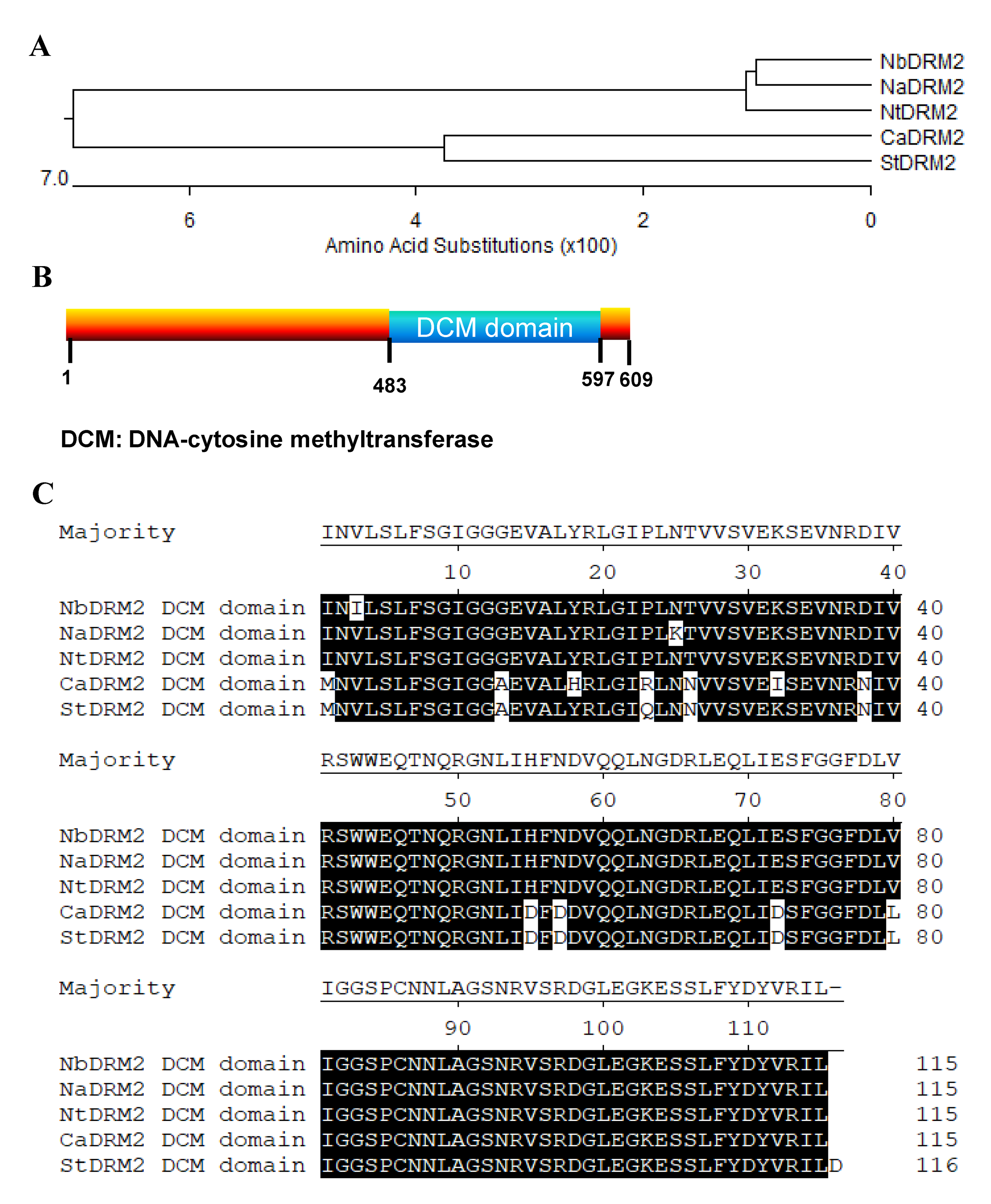

Supplement: S1 Fig — (A) Phylogenetic analysis of NbDRM2 homologues from different species based on the amino acid sequences using Clustal W from MegAlign software. (B) Schematic representation of NbDRM2 deduced from the SMART online software. (C) Sequence alignment of the catalytic DNA methyltransferase domain of NbDRM2 and orthologues using Meglign software. (TIF) [file ppat.1008829.s002.tif]

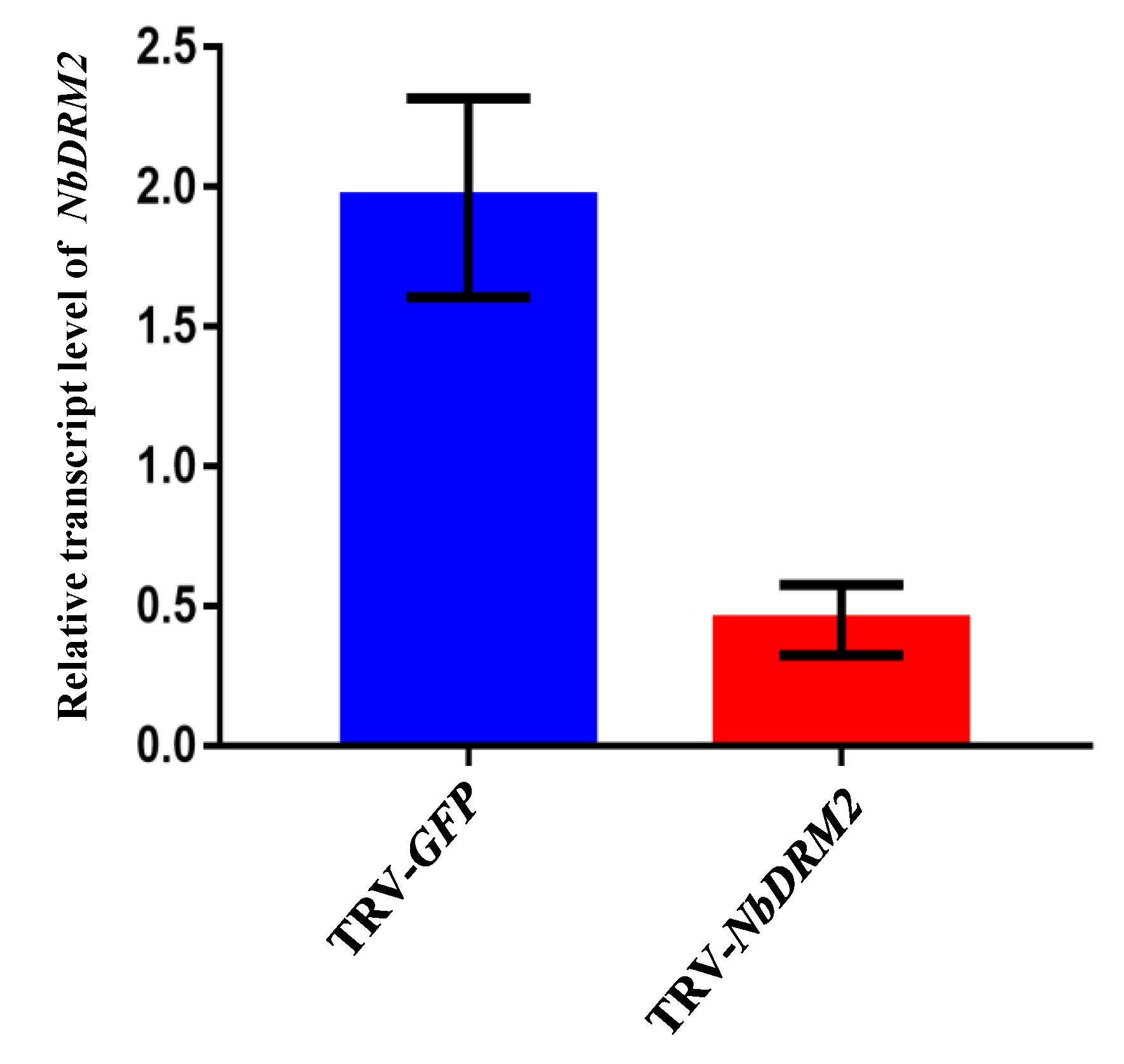

Supplement: S2 Fig — Relative accumulation level of NbDRM2 transcripts is normalized to the actin transcript. Error bars represent standard deviation of three biological replicates. (TIF) [file ppat.1008829.s003.tif]

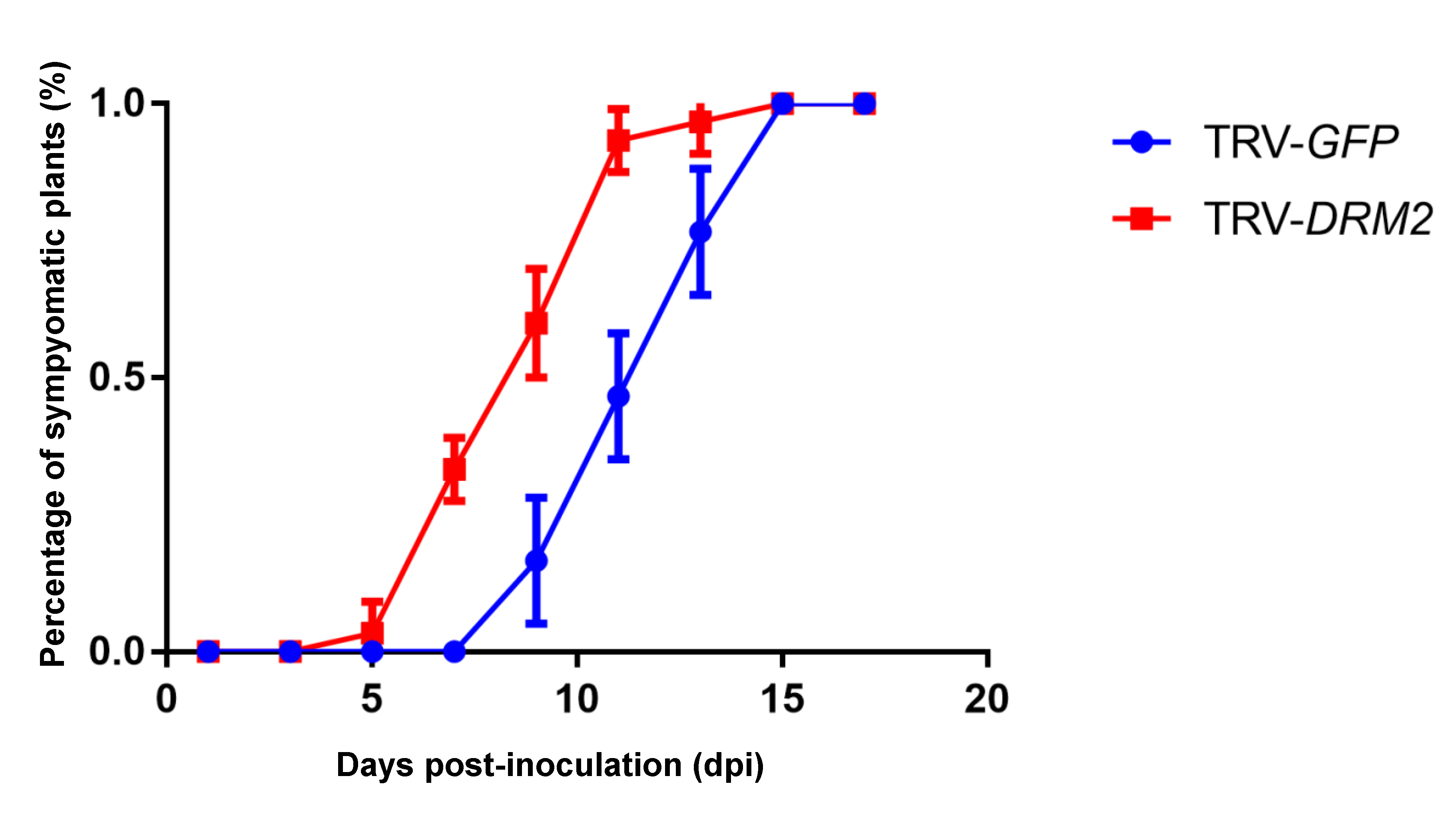

Supplement: S3 Fig — Red and blue curves represent the time course of TLCYnV symptom development in NbDRM2-silenced (TRV-DRM2) and mock (TRV-GFP) N. benthamiana plants, respectively. X-axis indicates days post-inoculation (dpi); Y-axis represents the percentage of symptomatic N. benthamiana plants. Over 60 N. benthamiana plants were used in this experiment. Error bars represent standard deviation of three biological replicates. (TIF) [file ppat.1008829.s004.tif]

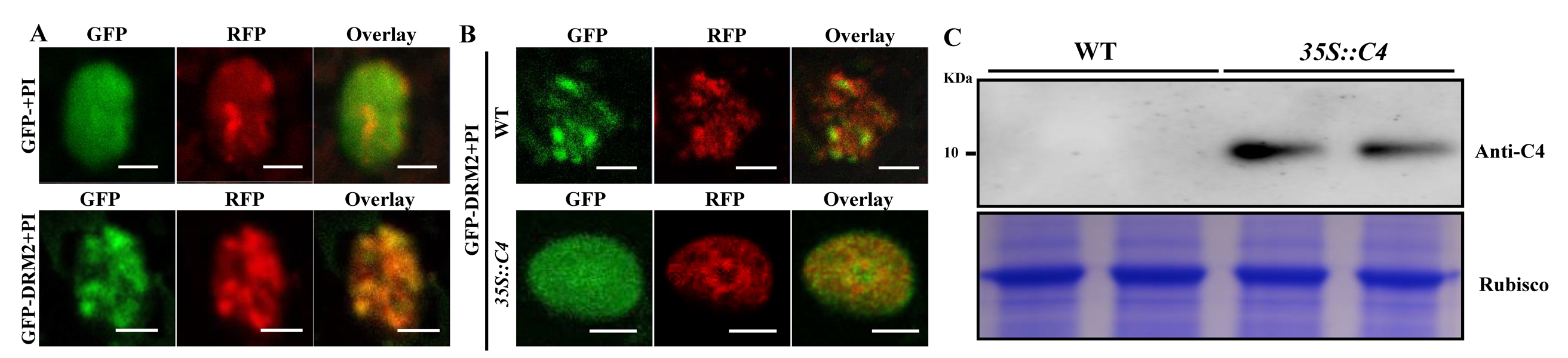

Supplement: S4 Fig — (A) Nuclear distribution of NbDRM2 in isolated nuclei; (B) TLCYnV C4 influences the NbDRM2 nuclear distribution pattern in isolated nuclei. Chromosomal DNA was stained by propidium iodide (PI). Scale bar = 20 μm. (C) Immunoblot analysis of TLCYnV C4 accumulation in 35S::TLCYnV C4 transgenic N. benthamiana plants. Two independent transgenic lines were used. Rubisco was used as loading control. (TIF) [file ppat.1008829.s005.tif]

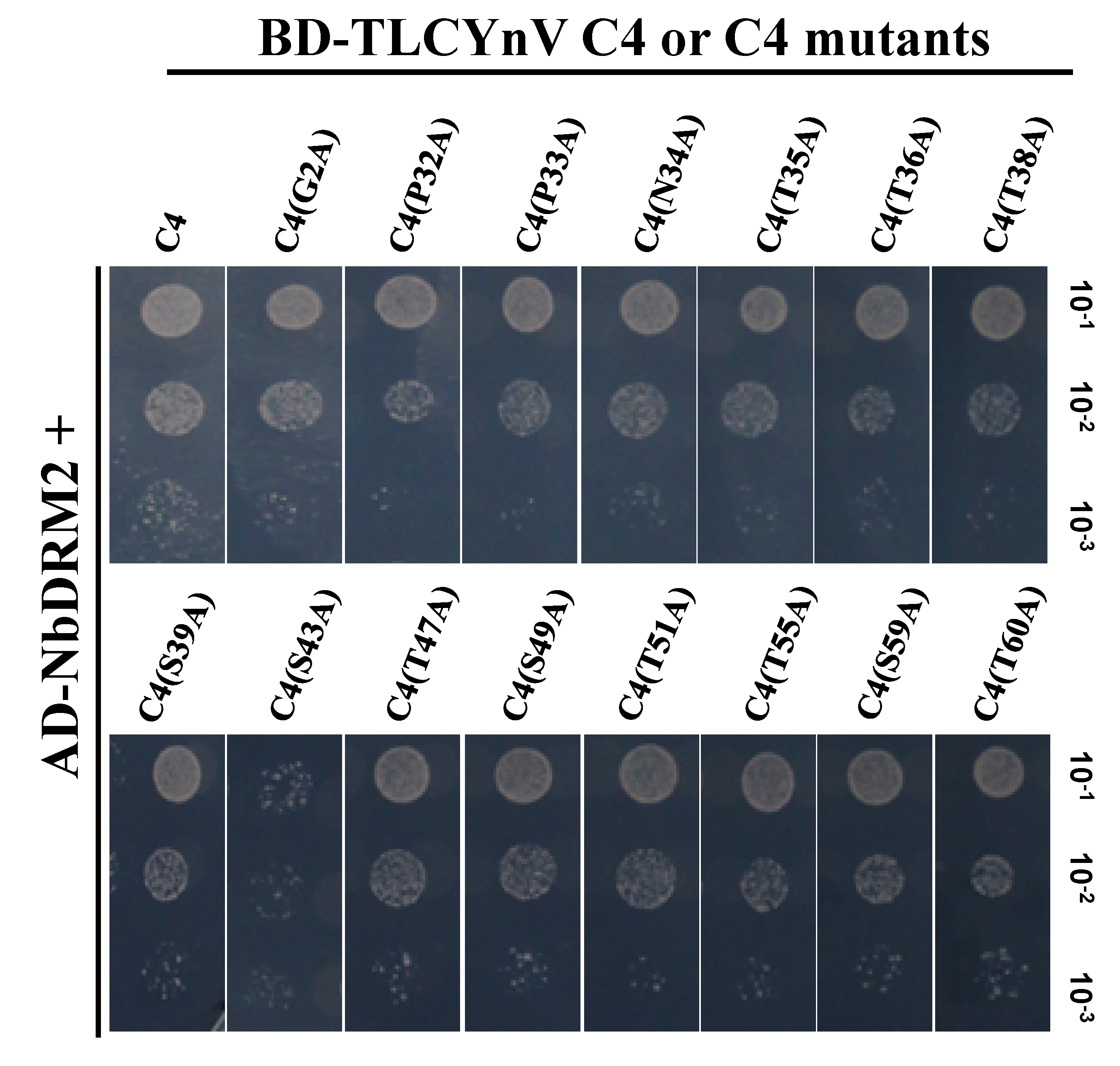

Supplement: S5 Fig — The yeast strain Gold co-transformed with the indicated plasmids was subjected to 10-fold series dilution, and grown on a SD/-Leu/-Trp/-His medium. (TIF) [file ppat.1008829.s006.tif]

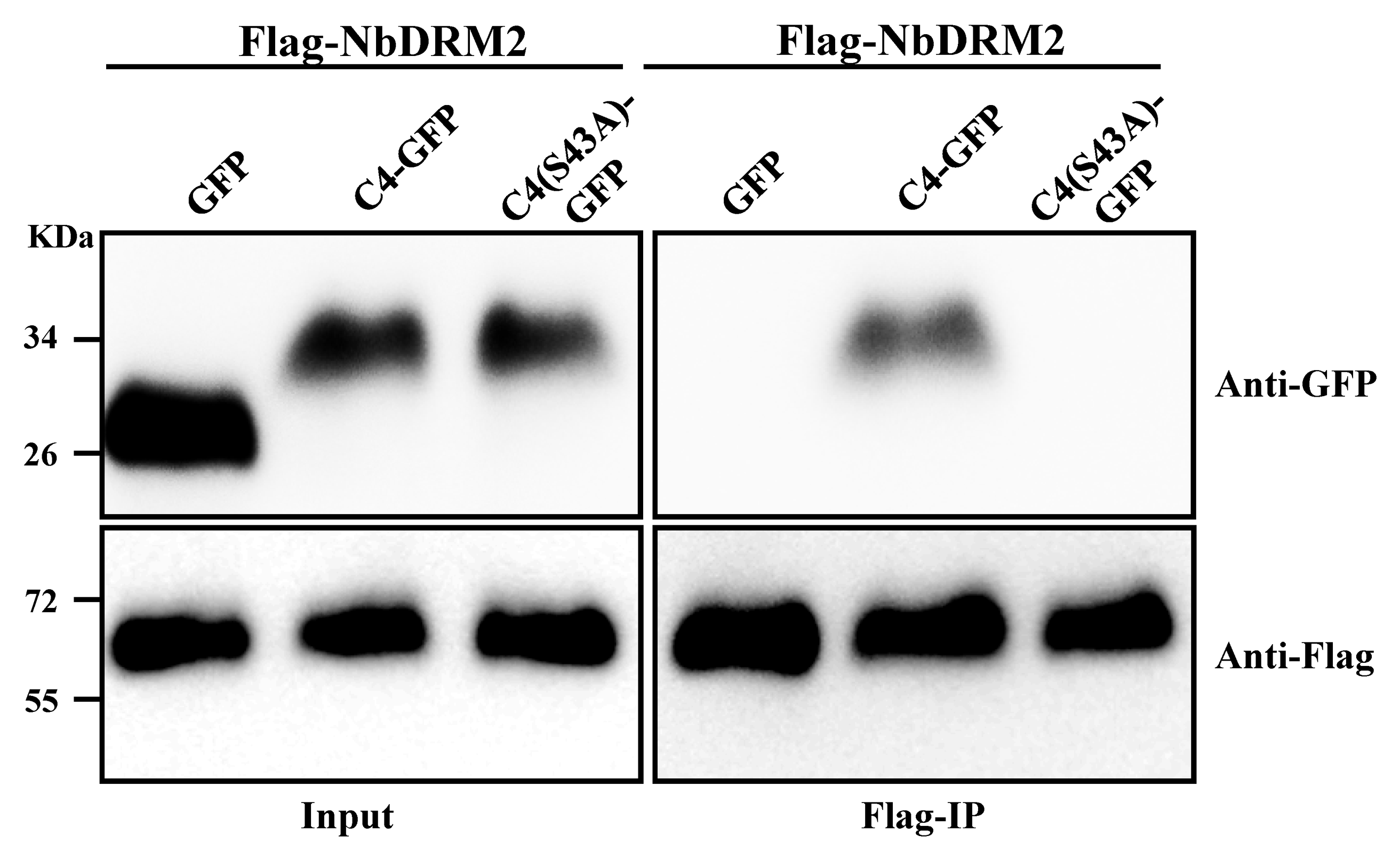

Supplement: S6 Fig — Leaves co-expressing Flag-NbDRM2 with GFP, TLCYnV C4-, or TLCYnV C4(S43A)-GFP were harvested at 2 dpi for co-immunoprecipitation (Co-IP) assays. Immunoblot analysis was conducted with antibodies specific to detect the indicated proteins. (TIF) [file ppat.1008829.s007.tif]

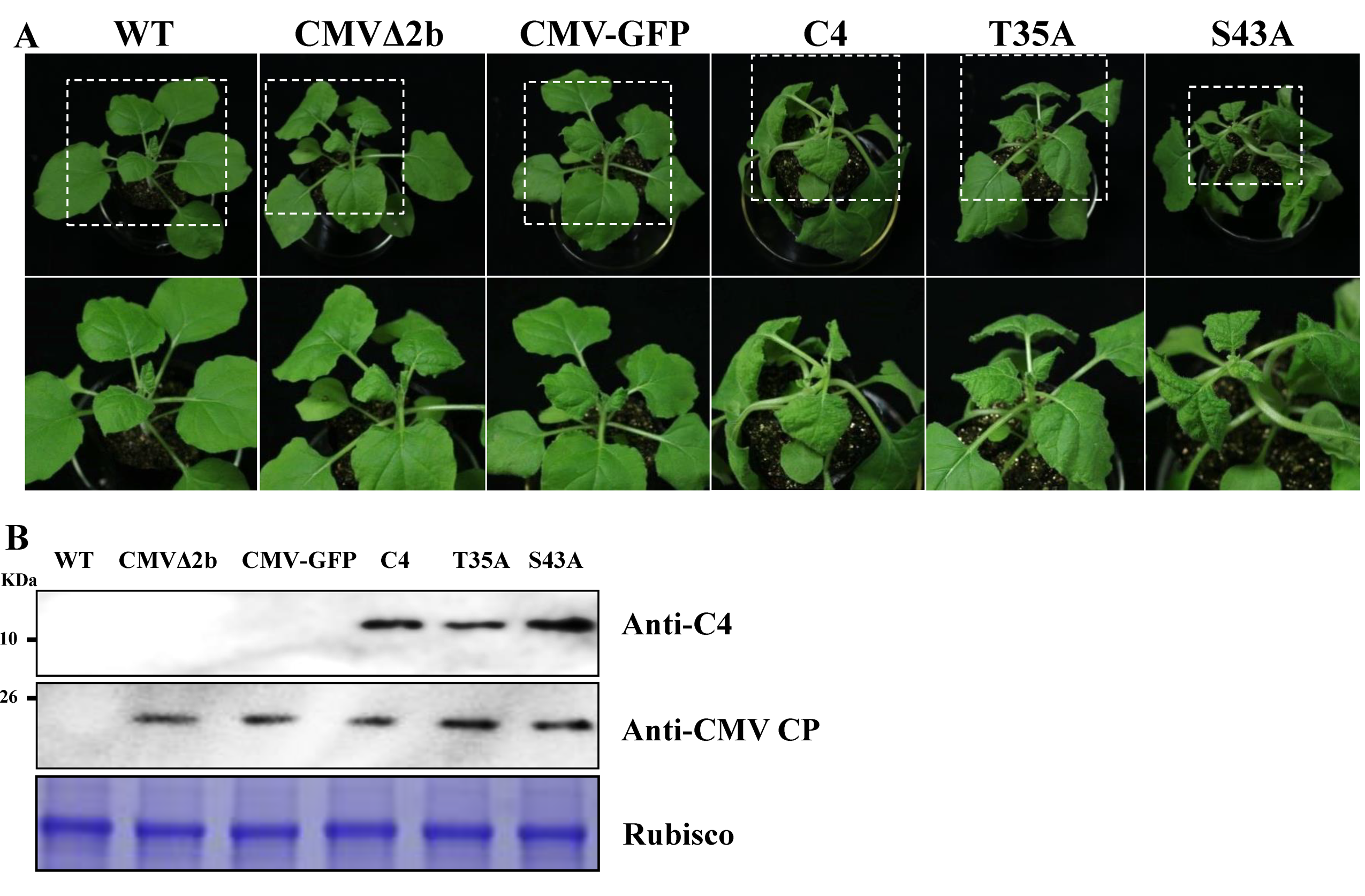

Supplement: S7 Fig — (A) Phenotype of N. benthamiana plants expressing TLCYnV C4 or C4 mutants systemically by using a CMV-based vector. The TLCYnV C4 mutant [C4(T35 A)], which does not induce viral symptoms, is used as control. Photographs were taken at 8 days post-inoculation. (B) Accumulation of TLCYnV C4 or C4 mutants in leaves of N. benthamiana plants under different treatments determined by Western blot using antibodies specific to the indicated proteins. (TIF) [file ppat.1008829.s008.tif]

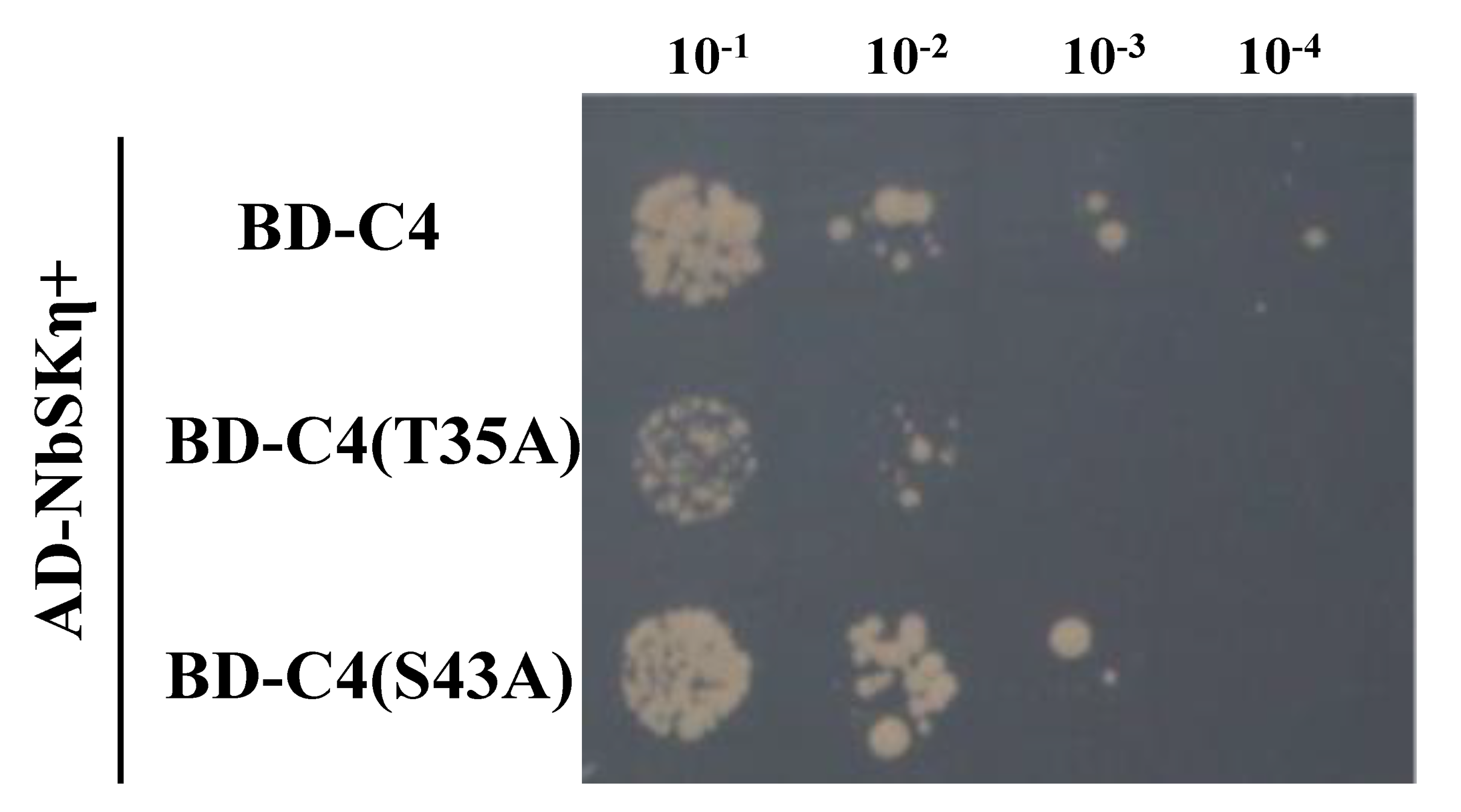

Supplement: S8 Fig — The yeast strain Gold co-transformed with the indicated plasmids was subjected to 10-fold series dilution, and grown on a SD/-Leu/-Trp/-His/-Ade medium. (TIF) [file ppat.1008829.s009.tif]

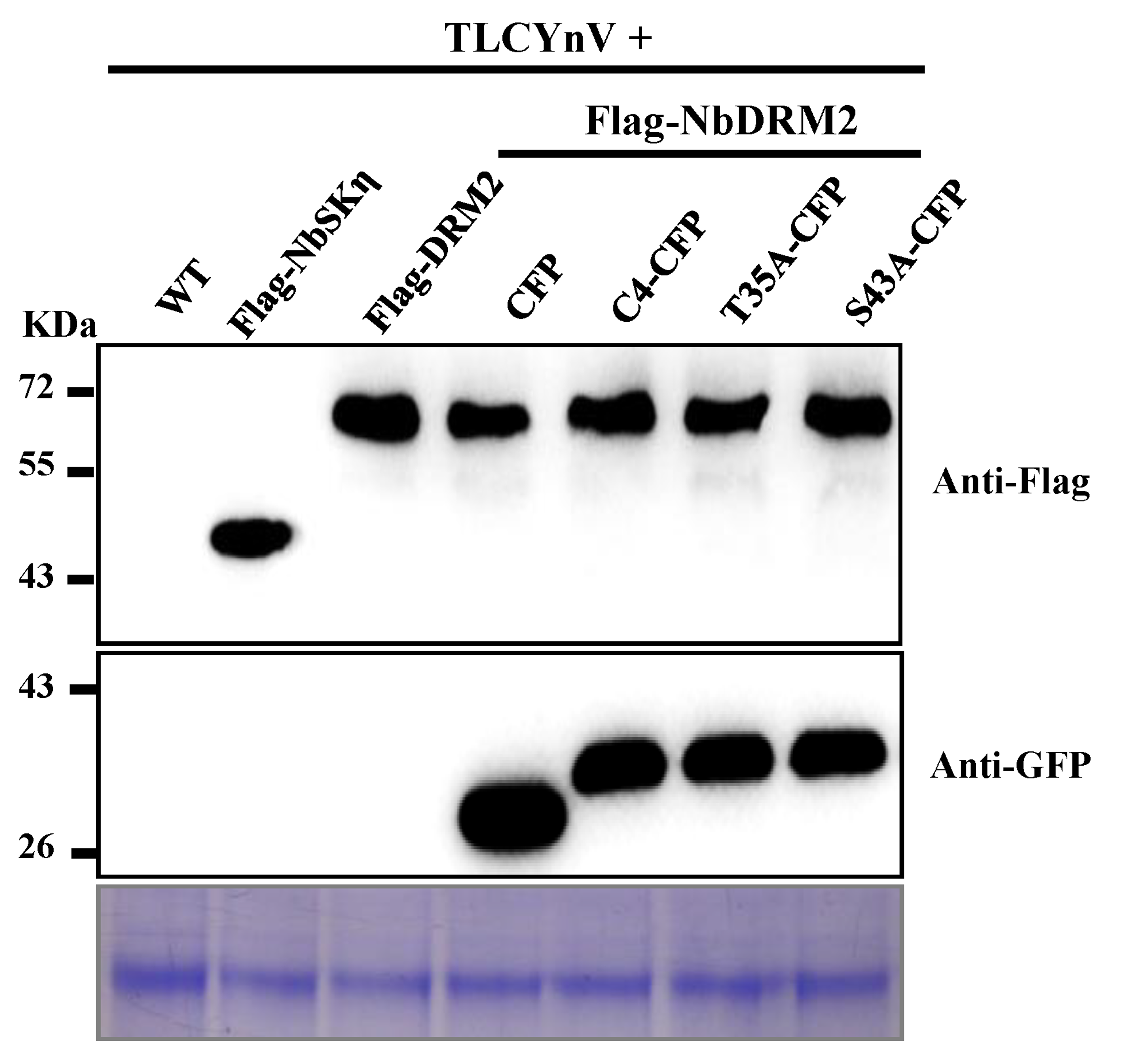

Supplement: S9 Fig — C4-, C4 mutants-GFP and Flag-NbDRM2 proteins were detected using GFP and Flag specific monoclonal antibodies. Rubisco was used as the loading control. (TIF) [file ppat.1008829.s010.tif]

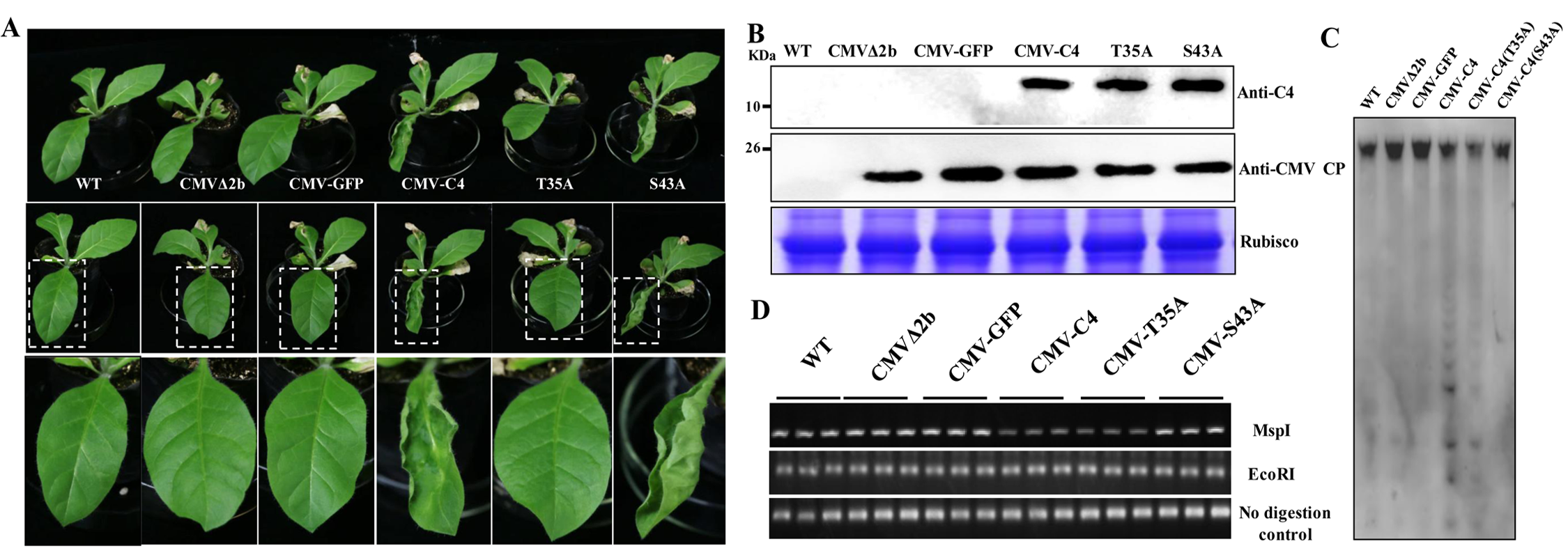

Supplement: S10 Fig — (A) Phenotype of N. tabacum plants expressing TLCYnV C4 or C4 mutants by using a CMV-based vector. Photographs were taken at 12 days post-inoculation. (B) Accumulation of TLCYnV C4 and C4 mutants in N. tabacum leaves under different treatments determined by Western blot using the antibodies specific to the indicated proteins. (C) Southern blot analysis of the methylation level of NtGRS1.3. Genomic DNAs from N. tabacum plants expressing TLCYnV C4 and C4 mutants were digested with MspI, then resolved in a 1.5% agarose gel. (D) Detection of the methylation level of NtGRS1.3 by using methylation-sensitive PCR. Genomic DNA was digested with MspI and then used as template for PCR. Undigested DNA was used as the loading control. (TIF) [file ppat.1008829.s011.tif]

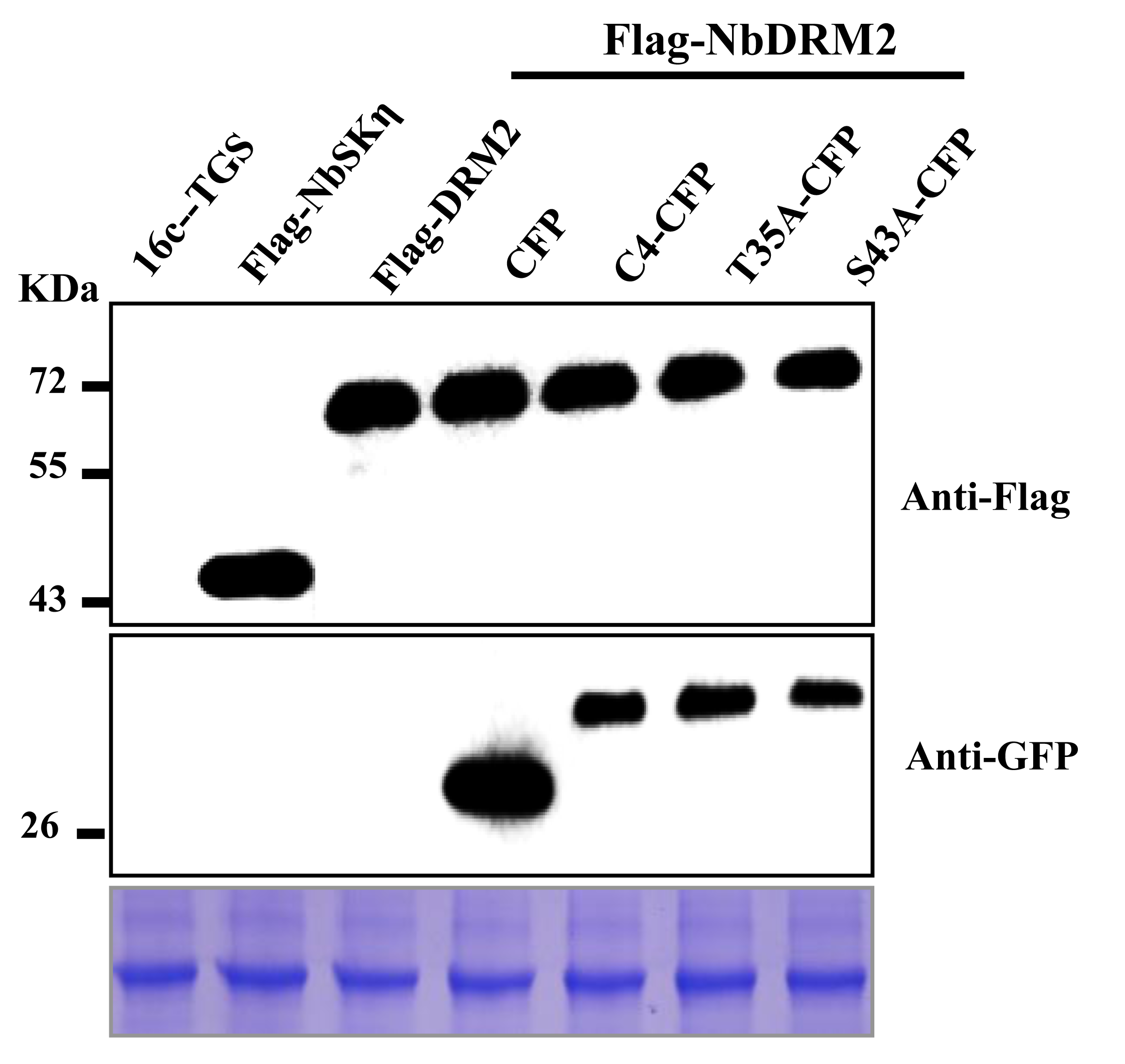

Supplement: S11 Fig — TLCYnV C4-, C4 mutants-GFP and Flag-NbDRM2 proteins were detected using monoclonal antibodies specific for GFP and Flag. Rubisco was used as the loading control. (TIF) [file ppat.1008829.s012.tif]

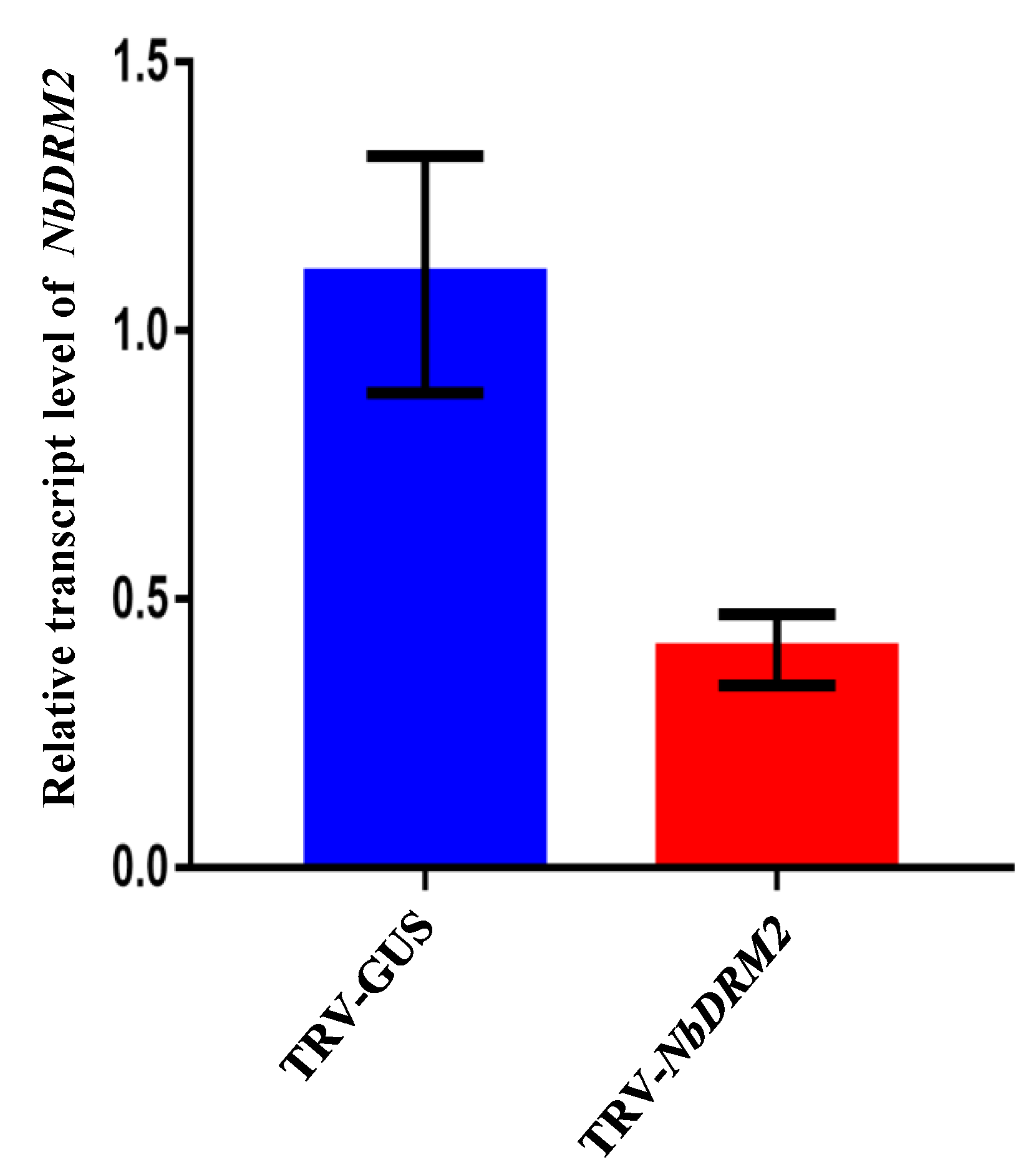

Supplement: S12 Fig — Relative accumulation of NbDRM2 transcripts is normalized to the actin transcript. Error bars represent standard deviation of three biological replicates. (TIF) [file ppat.1008829.s013.tif]
